# TRP channels and cancer modulation: a voyage beyond metabolic reprogramming, oxidative stress and the advent of nanotechnologies in targeted therapy

**DOI:** 10.1186/s13046-025-03495-4

**Published:** 2025-08-14

**Authors:** Marialaura Giannaccari, Chiara Florindi, Nora Bloise, Francesco Moccia, Francesco Lodola, Livia Visai

**Affiliations:** 1 Molecular Medicine Department (DMM), Centre for Health Technologies (CHT), Unità Di Ricerca (UdR) INSTM, Operative Unit (OU) of the Interuniversity Center for the Promotion of the 3Rs Principles in Teaching and Research (Centro 3R), University of Pavia, Pavia, 27100 Italy; 2 Department of Biotechnology and Bioscience, University of Milano-Bicocca, Milan, 20126 Italy; 3 UOR6 Nanotechnology Laboratory, Department of Prevention and Rehabilitation in Occupational Medicine and Specialty Medicine, Istituti Clinici Scientifici Maugeri IRCCS, Via Maugeri 4, Pavia, 27100 Italy; 4 Dipartimento Di Medicina E Scienze Della Salute “V. Tiberio”, Università Degli Studi del Molise, Campobasso, 86100 Italy

**Keywords:** TRP channels, Cancer, Metabolic reprogramming, Oxidative stress, Nanotechnologies

## Abstract

Transient receptor potential (TRP) channels are a large family of non-selective cation channels that play critical roles in cellular homeostasis and signal transduction. Recent investigations have clearly highlighted their involvement in cancer biology, particularly in the regulation of cancer metabolism. Unlike normal cells, cancer cells tend to favour the energy inefficient glycolytic pathway over the more effective oxidative phosphorylation process. TRP channels are involved in critical steps of cancer-related metabolic reprogramming by influencing intracellular Ca^2+^ signaling. Their dysregulation can intensify oxidative stress, thereby promoting oncogenic transformation and tumor progression. The intricate interplay between TRP channels, metabolic reprogramming and oxidative stress promotes cancer cell progression and resistance to treatment. This review highlights the crucial role of TRP channels in tumorigenesis. It examines how TRPM7 and TRPM8 contribute to metabolic reprogramming by its involvement in glycolysis pathway. Additionally, it explores the involvement of TRPML1, TRPA1, TRPM2, and TRPV1 in modulating reactive oxygen species (ROS) levels within cancer cells, analyzing the ROS dual role in tumor modulation. The advent of nanotechnology, particularly through the utilisation of engineered nanoparticles, has facilitated the selective modulation of TRPA1, TRPM2, and TRPV1 channels. This technological breakthrough has paved the way for novel and more targeted anticancer treatment strategies. The integration of molecular insights with cutting-edge technological approaches holds great promise for the development of more effective and targeted cancer treatments.

**Graphical Abstract:**

The intricate interplay between TRP channels and cancer progression.

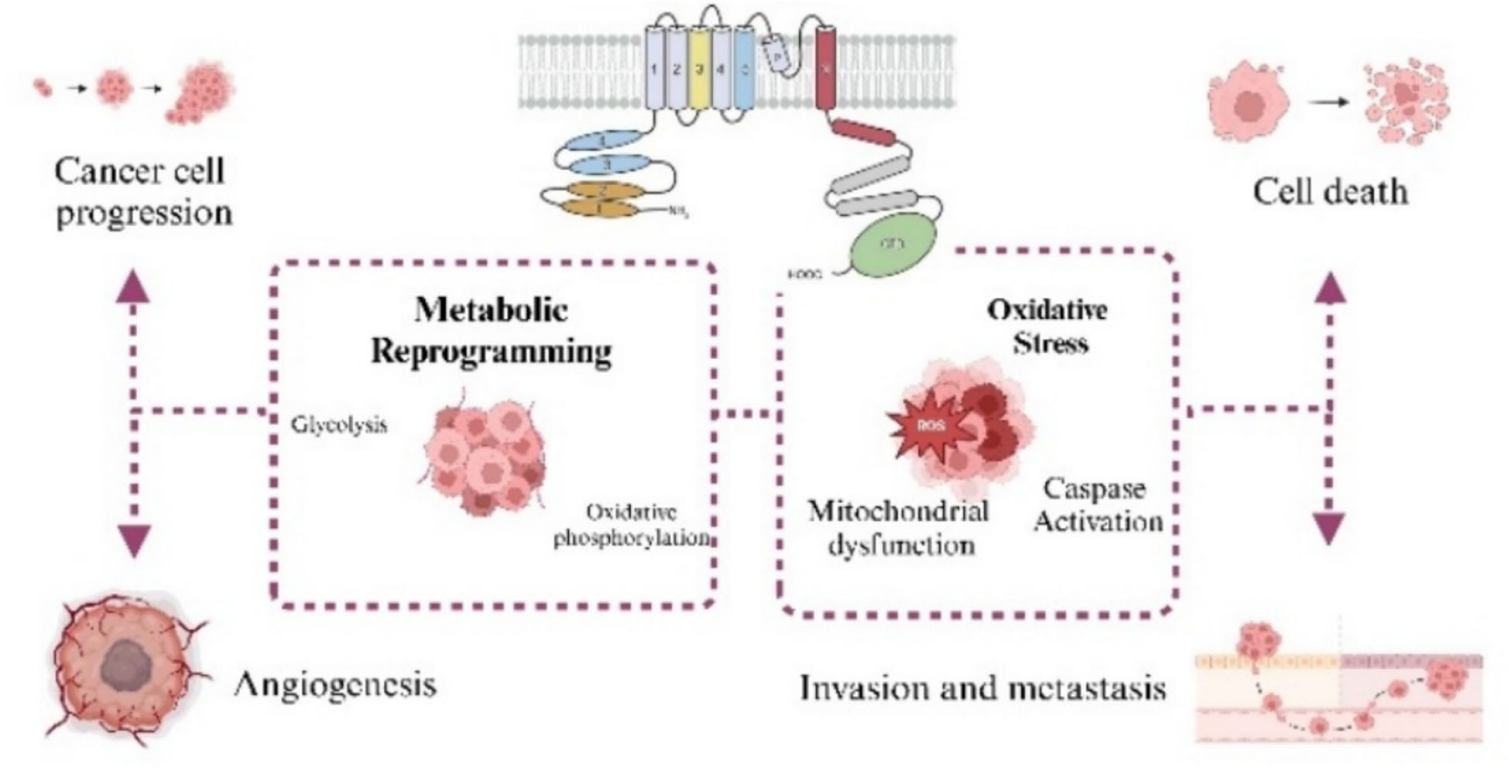

## Introduction

Cancer is a complex, multistage process that occurs when certain body cells proliferate out of control and metastasize, spreading to nearby tissues, and forming secondary malignancies in distant regions of the body [1]. Recent cancer statistics have indicated that, on average, 5,600 new cancer diagnoses and 1,700 cancer deaths occur each day. However, it is important to note that there has been a decline in cancer mortality rates over time, which can be attributed to improvements in cancer management and early-stage tumor detection [2]. Most cancer cases worldwide are attributed to seven redominant cancer types: lung cancer has the highest incidence and mortality in the world, followed by malignancies of the female breast, gastrointestinal tract, urological system, thyroid, cervix uteri, and non-Hodgkin lymphoma [2].

Due to the heterogeneity of the pathology, collaboration between researchers, doctors, patients and facilities is important to highlight cancer mechanisms, to find targeted therapies to minimize the collateral effects, and to discover new biomarkers for early diagnosis, which remains the most effective form of prevention [3].

In the impressive research effort to identify new therapeutic targets and understand the pathways underlying tumor progression, researchers have highlighted the role of TRP channels. TRP channels are a superfamily of non-selective cation-permeable (e.g., Na^+^, K^+^, and Ca^2+^) that have crucial role in a wide range of biological processes [4], including cancer development [5]. By altering intracellular Ca^2+^ levels or Ca^2+^-dependent signaling pathways, these channels support and exacerbate the growth of cancer, contributing in carcinogenesis and metastasis [6].

It has been demonstrated that TRP channels are related to all cancer hallmarks [7]. In particular, they were found to play a critical role in the progression of colon, breast, ovarian and bladder cancers [8]. The process of epithelial–mesenchymal transition (EMT), cytoskeletal remodeling, and invasive behaviour are modulated by Ca^2+^-dependent signaling pathways, including MAPK, PI3K/AKT, and MMP activation. These pathways are regulated by TRP isoforms such as TRPV4, TRPM7, TRPC1, TRPC3, and TRPC6 [9]. Furthermore, there is evidence to suggest that TRP channels are implicated in various cell death mechanisms. For instance, TRPM2 has been shown to regulate inflammasome activation in pyroptosis, while TRPM7 has been found to mediate Ca^2+^ influx in necroptosis via MLKL signaling [10]. Collectively, these TRP channels orchestrate key intracellular processes that govern tumor progression, resistance, and metastasis. Some research evidenced how the modulation of TRP channels is involved in metabolic reprogramming, affecting different ion signaling pathways, like glycolysis [11]or hypoxia-inducible factor (HIF) degradation [12]. The importance of TRP channels in tumor progression is further underlined by the fact that the redox modification of free thiol groups is the most common way in which ROS can directly activate TRP channels [13]. The oxidation of redox-sensitive residues in TRP channels modulates the fate of malignant cells, along with other effects on the tumorigenic process [14], determining mitochondrial dysfunction and the activation of cell death process [15]. Based on this evidence, the focus on TRP channels has emerged as a promising strategy for the identification of new drug targets, potentially leading to the development of more effective therapies. In this scenario, the advent of nanotechnology has enabled the implementation of tumor therapeutic plans that target TRP channels and exacerbate processes of cell death and mitochondrial dysfunction [16]. The growing attention to TRP channels in the field of oncology is revealed by a simple search of literature databases (PubMed), more than 600 research articles have been published until 2025 with increasing interest, especially in the last decade, in their involvement in cancer metabolism and oxidative stress. This review provides an in-depth and current analysis of the role of TRP channels in cancer, focusing particularly on their involvement in tumor-associated metabolic reprogramming and oxidative stress-mediated signaling pathways. After providing an overview of the fundamental characteristics, structure and physiological functions of TRP channels, we examine the emerging evidence supporting their pivotal role in cancer biology. This work aims to summarize how TRP channels function as sensors and mediators of oxidative stress in the tumor microenvironment. Finally, we highlight recent advances in nanotechnology, especially focused on nanoparticles and their potential to enable the selective modulation of TRP channels. We propose this integrated approach as a promising avenue for developing targeted cancer therapies. Combining insights from molecular oncology, redox biology and nanomedicine, this review emphasises the potential of TRP channels as biomarkers and therapeutic targets in cancer. It also proposes a dual conceptual framework addressing metabolic rewiring and oxidative stress as a foundation for future translational strategies.

## Overview of TRP channels: structure, functions, and versatility

**Fig. 1:**
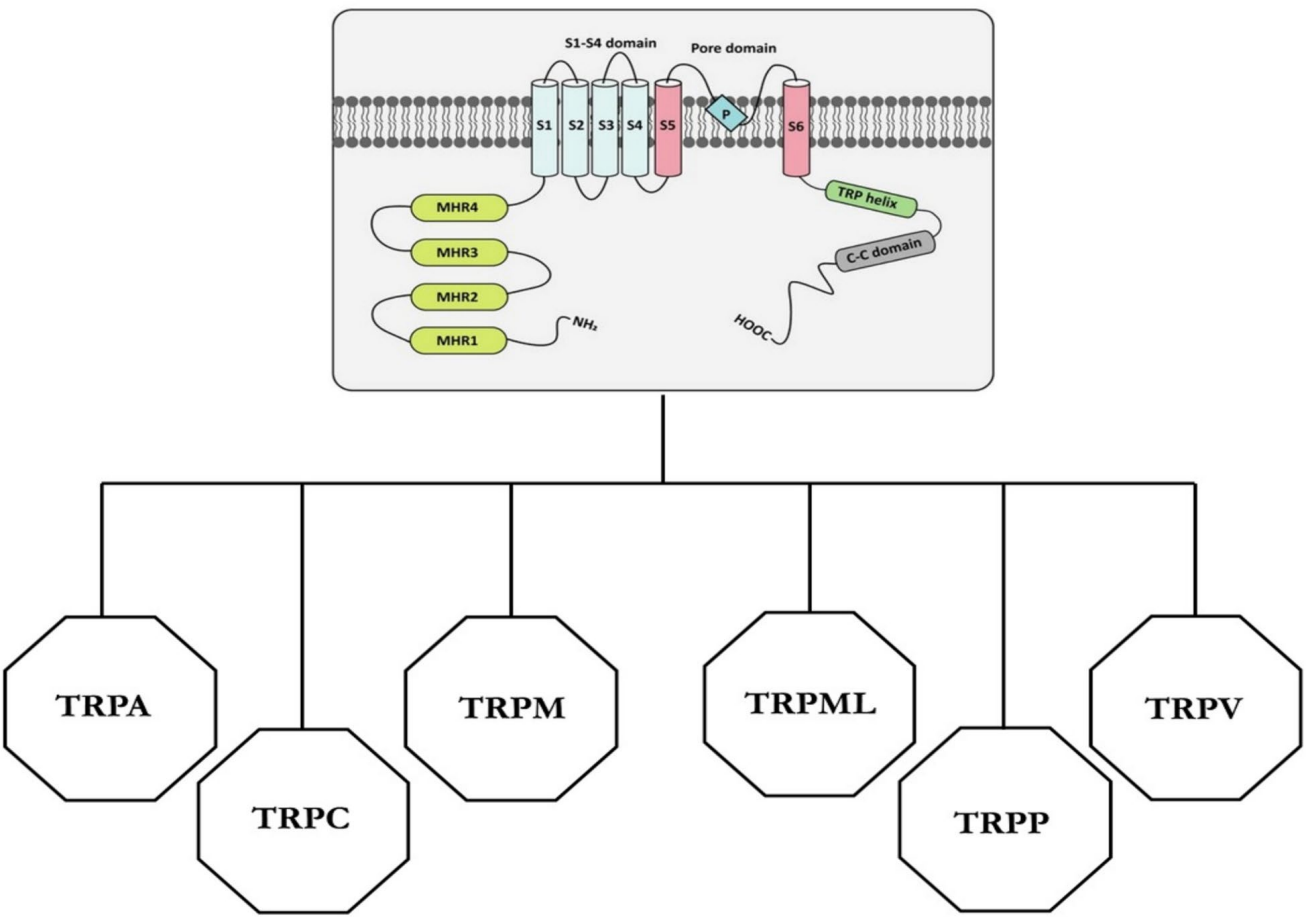
Schematic overview of TRP channel families. TRP channels are composed of six transmembrane segments (S1–S6), featuring a pore-forming loop between the S5 and S6 segments. Both the N- and C-termini are located intracellularly [22]. The cytoplasmic end of the S6 helix forms the lower gate, which controls the opening and closing of the channel to regulate cation flow. The S1–S4 segments may act as gating domains in response to ligand binding; however, the relatively low number of positively charged arginine residues in the S4 helix suggests limited voltage sensitivity of TRP channels. Regions outside the S5–S6 domain mediate interactions between subunits within the channel complex [23]. Based on structural homology, the 28 known TRP channel subunits are classified into six families: TRPA (ankyrin), TRPML (mucolipin), TRPM (melastatin), TRPC (classical or canonical), TRPV (vanilloid), and TRPP (polycystin) [23]. [Adapted from reference 23, Creative Commons Attribution 4.0]

The TRP channel superfamily can be divided into two main groups based on their properties (Fig. 1). The first subgroup includes TRPCs, TRPVs, TRPMs and TRPAs. They are composed of homo-or hetero-tetramers with each monomer consisting of six transmembrane α-elices followed by a TRP box, located after the S6 segment, which plays a key role in TRP channel gating. The second group includes TRPML and TRPP channels that do not contain the TRP helix [17–19]. The main differences within the TRP family are in the N and C domains, which are characterised by specific sites for protein interaction and regulatory motifs [5], and in the selectivity filter, which results in different cation permeabilities [20].

The gating properties of TRP channels are influenced by a variety of stimuli, such as heat, pressure, pH, temperature, osmotic changes, and mechanical forces, which may all converge on the activation of the same TRP isoform [18]. Therefore, TRP channels are regarded as multimodal sensors of both the extracellular and intracellular microenvironments [21, 22]. For example, TRPV1 is activated by heat or capsaicin, but is also sensitive to ROS [23], while TRPM8 responds to cold and menthol, but may also act as a Rap1 GTPase inhibitor [24].

In addition, TRP channels are versatile and respond to a wide range of signals, ensuring the Ca^2+^ influx necessary for many essential cellular functions, including metastatic progression [25, 26]. These channels are involved in Ca^2+^ influx through a process known as store-operated Ca^2+^ entry (SOCE). In this case, the depletion of intracellular Ca^2+^ stores is detected by the ER sensor STIM1, which activate TRP channels, such as TRPC1, on the plasma membrane, allowing Ca^2+^ influx from the extracellular space. In this scenario, TRPC1 assemblies with the Ca^2+^-selective plasma membrane channel, Orai1, and may also bring TRPC4 into the supramolecular signaling complex [27]. Another mechanism supporting Ca^2+^ entry across the plasma membrane is receptor-operated Ca^2+^ entry (ROCE), where TRP channels are activated by signaling pathways downstream of GPCRs or tyrosine kinase receptors, without requiring depletion of intracellular Ca^2+^ stores. For instance, TRPC3 and TRPC6 are activated by the second messenger, diacylglycerol (DAG) [28, 29], whereas TRPC4 and TRPC4 can serve as a coincidence detector for Gαi and Gq-dependent increases in intracellular Ca^2+^ concentration ([Ca^2+^]_i_) [30]. Other TRP channels, like TRPM2, are more likely to function as ROS sensors, being directly activated by hydrogen peroxide (H_2_O_2_) as well as by cytosolic signals such as ADP-ribose or NAD⁺, that are typically produced in response to oxidative stress [14, 31].

The physiopathological relevance of TRP channels is evidenced by their involvement in ion exchange, which consequently affected downstream pathways such as: mitogen-activated protein kinase (MAPK), transforming growth factor (TGF-β) signaling pathway, nuclear factor kappa-B (NF-κB) pathway, and AMP-activated protein kinase (AMPK) pathway [32].

Consequently, TRP channels pose a persistent challenge within the domain of biomedical research. Despite considerable progress in understanding their diverse roles, many aspects of their activation, modulation, and involvement in both normal physiological processes and pathological conditions remain incompletely elucidated. Further research into the molecular mechanisms that govern TRP channel function is required to enhance our current understanding. By elucidating the mechanisms through which these channels contribute to disease progression and tissue homeostasis, researchers can identify new targets for drug development, which may result in the development of innovative treatments and enhance human health.

## TRP channels in cancer progression

Each form of cancer has distinct clinical characteristics, molecular indicators, and a gene profile particular to the disease [33]. However, all forms of cancer share several characteristics, referred to as the"cancer hallmarks,"which were outlined in 2000 [34]. These hallmarks include evading apoptosis, self-sufficiency in growth signals, sustained angiogenesis, insensitivity to anti-growth signals, limitless replicative potential, tissue invasion and metastasis [34]. These hallmarks were further elaborated in 2011 with the concept of reprogramming cellular metabolism and evading immune destruction [35].

Numerous studies have highlighted the pivotal roles of ion-permeable channels in various aspect of cancer, including cell proliferation, resistance to apoptosis, invasion, and drug resistance [25]. Alterations in the expression and/or function of specific ion channels have been strongly associated with cancer, leading to the coining of the term"oncochannels"[36]. Recently, there has been increased interest in TRP channels, which are overexpressed and dysregulated in various carcinomas, such as TRPC3 in ovarian cancer [37], TRPC6 in hepatocellular carcinoma [38], TRPM7 in breast cancer [39, 40] and TRPM8 in prostate cancer [41]. Although the precise molecular mechanisms through which these channels contribute to cancer remain incompletely understood, evidence suggests that TRP channels influence tumor progression. For instance, TRP channels activity regulates actin filament dynamics and integrin function, which are essential for cell migration and invasion [42]. Furthermore, TRP channels play a crucial role in balancing pro-survival and pro-apoptotic signals, thereby facilitating cancer cell resistance to cell death [9].

Recent studies have elucidated the crucial role of TRP channels, specifically TRPC3 [43],TRPC5 [44, 45],TRPV4 [46] TRPML3 [47], in regulating the release, cargo content, and downstream effects of cancer-derived exosomes within the tumor microenvironment (TME).

TRPC3-mediated Ca^2+^ influx promotes the formation of multivesicular bodies (MVBs) and their fusion with the plasma membrane, which are key steps in exosome secretion. In SKOV3 ovarian cancer cells, Padbury et al. demonstrated that the pharmacological activation of TRPC3 significantly increased extracellular vesicle (EV) release, while its inhibition suppressed EV output [43]. These exosomes were shown to carry oncogenic proteins and signaling molecules, contributing to enhanced tumor proliferation and intercellular communication. This positions TRPC3 as a potential therapeutic target to disrupt pathological exosome-mediated signaling in cancer [43].

TRPC5 promotes chemoresistance in breast cancer through the release of exosomes enriched with TRPC5 protein and associated drug-efflux machinery [44]. These vesicles are taken up by adjacent cancer cells, thereby spreading resistance traits via the modulation of the NFATc3 transcriptional pathway and Ca^2+^-dependent signaling cascades [48]. Additionally, TRPC5-mediated exosome secretion is linked to the activation of P-glycoprotein and Wnt/β-catenin signaling, both of which contribute to enhanced tumor survival and intercellular communication under therapeutic pressure [49].

TRPV4 is downregulated in tumor endothelial cells due to exosomal signals derived from malignant cells, leading to disrupted vascular architecture and abnormal angiogenesis [46]. This effect is mediated by the RhoA/ROCK–YAP–VEGFR2 signaling axis and can be reversed by inhibiting exosome release or pharmacologically restoring TRPV4 function [50]. Moreover, in cancer cells, TRPV4 facilitates metastatic progression by modulating actomyosin contractility and cell stiffness through Ca^2^⁺-dependent activation of the AKT–E-cadherin pathway, ultimately enhancing transendothelial migration and the invasive potential of tumor cell [51]. These processes are tightly associated with cytoskeleton remodeling and vesicle trafficking that favor the formation and directional release of pro-metastatic exosomes [52].

TRPML3, predominantly localized to late endosomes and lysosomes [19], regulates intracellular vesicle fusion and exosome biogenesis by controlling Ca^2+^ release from endolysosomal stores [53]. Loss or dysfunction of TRPML3 disrupts MVB maturation and impairs exosomal cargo sorting, particularly affecting the trafficking of signaling molecules involved in tumor proliferation and immune evasion [47]. Through its role in autophagy-lysosomal pathways and membrane recycling, TRPML3 also influences the secretion of exosomes carrying key regulators of tumor metabolism and microenvironmental remodeling [47].

Collectively, these findings reveal that TRP channels function as critical modulators of exosome dynamics in cancer, interfacing with signaling networks that drive tumor progression, angiogenesis, and metastasis. Their dual involvement in vesicle trafficking and signaling crosstalk underscores their potential as therapeutic targets in oncology.

Firstly, natural products have garnered significant attention for their potential to modulate TRP channels activity, offering promising avenues for targeted and low toxicity oncotherapeutic strategies. Capsaicin, the pungent compound found in chili peppers, is a well-characterized TRPV1 agonist that induces Ca^2+^ overload, ultimately triggering apoptotic cell death in prostate and breast cancer cells [54]. Curcumin, a polyphenol derived from *Curcuma longa*, has been shown to modulate TRPM8 channels, thereby inhibiting prostate cancer cell proliferation and inducing cell cycle arrest [41]. Similarly, resveratrol has been reported to downregulate TRPM2 expression, disrupting oxidative stress–mediated survival pathways in glioblastoma cells [55]. These findings underscore the potential of natural TRP modulators to influence cancer cell viability through ion channel–dependent mechanisms, highlighting their utility as adjuvants in combinatorial cancer therapies. Thanks to their importance, many TRP channels have therefore been investigated as novel therapeutic targets and prognostic indicators in various cancer types [44, 45]. Within the TRPC subfamily, compounds such as Pico145 (C31) have emerged as highly potent and selective inhibitors of TRPC1/4/5 channels [58]. Structural studies confirm that Pico145 binds to a conserved lipid-interaction pocket in TRPC5, offering a solid basis for rational drug design [58]. Comparable small molecules, including ML204 and AC1903, demonstrate micromolar to submicromolar inhibition of TRPC4/5, providing a toolbox for dissecting channel-specific roles in oncogenic calcium signaling [59]. TRPM8 activator D3263, a clinical Phase 1 dose escalation study (NCT00839631), helped patients with prostate cancer stabilize their condition [46]. Also, WS-12, another TRPM8 activator, could be utilized as a prostate cancer diagnostic marker [40]. An additional example is the Phase 1 trial of SOR-C13 (NCT01578564). This TRPV6 inhibitor reduces cancer growth and spread that is mediated by Ca^2+^, for instance in small cell lung cancer (SCLC) [47]. Finally, hydrogen sulphide (H_2_S) can be released by H_2_S donors, including S-propargyl-cysteine, sodium hydrosulfide, and GYY4137 [192], to stimulate TRPV1, thereby inhibiting metastatic colorectal carcinoma cancer cell proliferation [193].

## TRP channels in cancer metabolic reprogramming

Cancer cells undergo a metabolic reprogramming process to support the energy, biosynthetic and redox requirements of the tumor [48]. This metabolic reprogramming, known as “Warburg effect”, it is characterised by increased glucose consumption and lactate production, which is influenced by gene and protein levels, as well as tumor-host cell interactions [49]. It is notable that cells possess a wide range of ion channels, particularly TRP channels, which meticulously regulate intracellular signaling [5]. These channels may function as pivotal lipid-sensitive regulators of cellular metabolism by integrating lipid-derived signals within the membrane microenvironment [64]. Activation of TRPV1 by dietary bioactive compounds, such as capsaicin, has been demonstrated to reduce lipid accumulation through upregulation of fatty acid oxidation genes and stimulation of lipolysis [65]. In parallel, TRPM2 contributes to maintaining mitochondrial redox homeostasis and prevents lipid-induced inflammation by modulating Ca^2+^ signaling and mitochondrial function [65]. Moreover, during ferroptosis, a regulated iron-dependent form of cell death characterized by lipid peroxidation, TRP channel activation exacerbates intracellular Ca^2^⁺ overload, driving downstream cell death pathways. Notably, both TRPV1 and TRPA1 are activated by electrophilic lipid peroxidation products, indicating a direct molecular link between dysregulated lipid metabolism and TRP channel function [62, 63]. Thus, lipid peroxidation-mediated modulation of TRP channels constitutes a critical intersection between membrane lipid remodeling, ion channel physiology, and metabolic regulation, with profound implications for diseases associated with oxidative stress, metabolic dysfunction, and ferroptotic cell death [66].

These channels have been shown to facilitate cancer cells'adaptation to the tumor microenvironment, including nutrient deprivation and hypoxia. In this context, evidence has emerged demonstrating the capacity of TRP channels to modulate HIF-1α activity [50, 51], affect the AMPK pathway [52] and the activity of glucose transporters (GLUTs) [53].

Some recent studies have highlighted the crucial role of two types of TRP channels, TRPM7 [6, 7, 67] and TRPM8 [71], in regulating cell metabolism in certain tumor types.

In the human body, the TRPM7 protein is widely expressed [73]. It is a peculiar channel characterized by both ion channel and enzymatic activity and is, therefore, known as chanzyme. Specifically, it acts as a serine-threonine protein kinase (α-kinase) thanks to the presence of autophosphorylation residues. The channel represents a master regulator of the cellular balance of divalent cations and mediates the uptake of Zn^2+^, Mg^2+^ and Ca^2+^, which are catalytic and structural cofactors of numerous enzymes and are major regulators of signaling molecules, DNA stability, cell cycle and transcription factors [55]. The expression and/or the function of TRPM7 has been shown to be upregulated in many malignant cancer types, including colorectal carcinoma, prostate cancer, ovarian cancer, and breast cancer [56].

Another key player of metabolic reprogramming is TRPM8, a member of the melastatin subfamily. This channel is permeable to both monovalent and divalent cations, including Na^+^, K^+^ and Ca^2+^, and it is activated by low temperature and cooling agents such as menthol [57, 74]. TRPM8 is expressed in several tissues, including the breast, pancreas, bone and colon, and its overexpression has been associated with cancer hallmarks such as cell migration and invasion [75].

### Significance of TRPM7 and TRPM8 in cancer metabolic reprogramming

In this section, we discuss recent findings involving TRPM7 and TRPM8 in metabolic reprogramming in different tumor types (Table 1).

**Table 1:** Summary of recent findings on TRPM7 and TRPM8 channel in cancer metabolic reprogramming

TRP Channel	Type of tumor	End-Point	Ref.
TRPM7	Bladder cancer	The KO-TRPM7 cell lines decreased glycolytic related enzymes (HK2, ENO2, PDK1) and controlled the glucose metabolism through SLC2A3 expression, which encodes for glucose transporter 3 (GLUT3)	[11]
TRPM7	Ovarian cancer	TRPM7 silencing determined a metabolic reprogramming from glycolysis to OXPHOS Protein expression exhibited a decrease of glycolysis related enzymes (HK2 and PDK1) and an increase of OXPHOS related enzymes (IDH3B and UQCRC1)	[12]
TRPM7	Hepatocellular carcinoma	Hypoxia conditions determined TRPM7 overexpression and an increase of glycolytic related enzymes (PGM1, PGM2, PGM3 and PFKL). KO-TRPM7 cell lines showed a reduction of migration and metastasis in vitro and in vivo HCC models	[98]
TRPM8	Hepatocellular carcinoma	Gene and protein expression unveiled TRPM8 overexpression in HCC TRPM8 knockout inhibited HCC progression by interfering with mitochondrial function, through the releasing of Cyt C which activated Caspase 9 TRPM8 KO downregulated the expression SNORA55 by influencing chromosome structure and histone modifications	[102]

*Abbreviations: TRPM7* transient receptor potential cation channel subfamily M member 7, *KO* (Knock-out), *HK2* Hexokinase2, *ENO2* Enolase 2, *PDK1* Phosphoinositide-dependent kinase-1, *SLC2A3* Solute Carrier Family 2 Member 3, *GLUT3* Glucose transporter- 3, *OXPHOS* oxidative phosphorylation, *IDH3B* isocitrate dehydrogenase (NAD(+)) 3 non-catalytic subunit beta, *UQCRC1* ubiquinol-cytochrome c reductase core protein 1, *HCC* Hepatocellular carcinoma, *PGM1* phosphoglucomutase 1, *PGM2* phosphoglucomutase 2, *PGM3* phosphoglucomutase 3, *TRPM8* transient receptor potential cation channel subfamily M member 8, *Cyt C* Cytochrome c, *SNORA55* Small Nucleolar RNA, H/ACA Box 55

#### TRPM7

##### Bladder cancer

Bladder cancer (BC), also known as urothelial bladder cancer, accounts for 2.8% of all cancer deaths, according to Global Cancer Observatory (GLOBOCAN) [76]. There are several risk factors related to the onset of the disease including diet, smoking, environmental factors, diseases. The presence of the disease in its early stages poses a significant challenge in terms of effective treatment. The diagnosis of this type of tumor is made using a range of methods, including cystoscopy with biopsy, imaging techniques, urinary cytology, FISH, and urine-based protein markers [77]. Cystoscopy and urinary cytology are the current gold standards [78]. However, it should be noted that cystoscopy is an invasive procedure which may result in complications, including pain, bleeding, or infections, and may result in the failure to detect tumors in areas that are difficult to reach [79]. Urinary cytology, despite being non-invasive, simple and low-cost, exhibits limited sensitivity, particularly in the case of low-grade tumors [80]. The standard treatments for BC are twofold: the first is platinum-based chemotherapy, which is the first line of intervention for muscle invasive BC, which precedes cystectomy; and the second is Bacillus Calmette-Guerin for the treatment of non-muscle invasive BC [81]. Since the efficacy of these treatments are not optimal, new therapeutic strategies are currently under investigation, such as antibody conjugates, fibroblast growth factor receptor targeting, CAR-T therapy [60, 61, 82]. Thus, understanding the different pathways involved in tumor progression and tumor proliferation may be important to positively improve BC research.

**Fig. 2:**
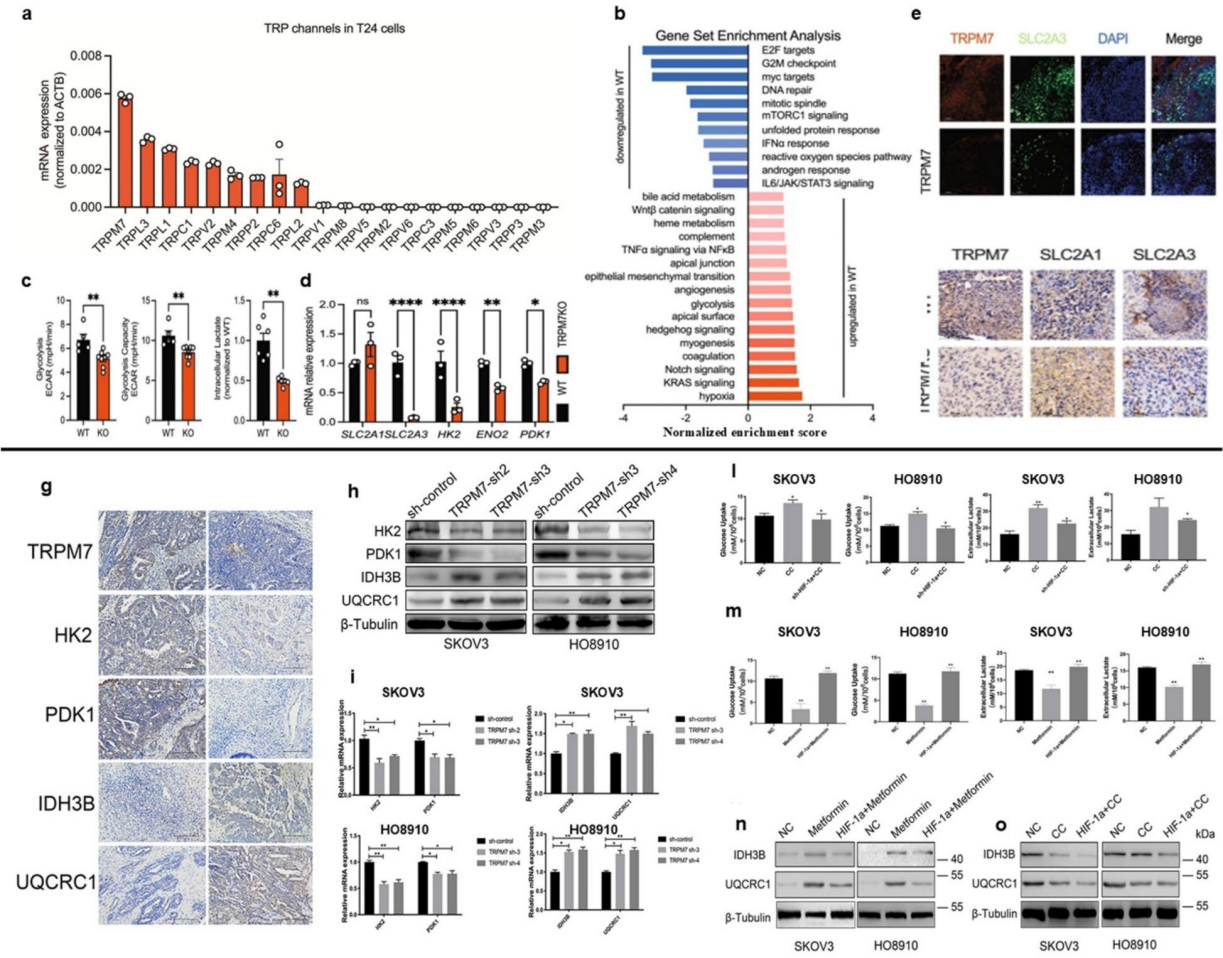
*TRPM7* role in metabolic reprogramming in bladder (**a**-**f**) and ovarian cancer (**g**-**o**). **a **RT-qPCR analysis of different TRP channels, data were normalized on β-actin (ACTB). The results revealed that TRPM7 was the most expressed. **b** Investigation of the hallmark gene sets in TRPM7KO versus WT T24 cells using gene set enrichment analysis (GSEA). **c** Extracellular acidification rates (ECAR) were used to quantify glycolysis in WT and TRPM7KO cells in both basal and response conditions to 10 mM glucose, 3 μM oligomycin, or 100 mM 2-deoxy-D-glucose (2-DG). Relative lactate within the cell in either TRPM7KO or WT cells. **d **RT-qPCR analysis confirming the reduced expression levels of glucose catabolic genes from RNA-seq in TRPM7KO cells.** e **TRPM7, SLC2A3, and DAPI immunofluorescence labelling in the xenograft tumor (scale bars, 100 μm).** f** TRPM7, SLC2A1, and SLC2A3 immunohistochemical staining of tumor tissue (size bars, 100 μm) [from a to f, *Creative Commons Attribution 4.0 International License*; [11]]. **g** IHC study of the expression of TRPM7, HK2, PDK1, IDH3B, and UQCRC1 in human ovarian cancer and non-tumor tissues. The scale bar,50 μm. **h–i** WB and RT-qPCR evaluations of the expression of HK2, PDK1, IDH3B, and UQCRC1 in SKOV3 and HO8910 cell lines. **l-m** Evaluating glucose uptake, lactic acid generation, and ECAR in SKOV3-sh-control, SKOV3-sh-TRPM7, HO8910-sh-control, and HO8910-sh-TRPM7 cells with or without treatment with Compound C (CC) or metformin.** n-o** Western blot of AMPK phosphorylation, IDH3B, UQCRC1, HIF-1α, HK2, and PDK1 expression in the indicated cell groups [from g to o, *Creative Commons Attribution 4.0 International License* [12]]

In this scenario, one of the most recent investigations, which emphasize the role of TRPM7 in tumor metabolic pathway was conducted by Wu et its collaborators [11]. These researchers firstly unveiled that in contrast to other TRP channels, TRPM7 was the most overexpressed channel in T24 urinary BC cell line [6] (Fig. 2a). The focus of this work was to understand how channel could affect glycolytic pathway. In KO-TRPM7 cells, gene set enrichment analysis revealed the downregulation of glycolysis related genes (Fig. 2b). This data was confirmed by oxygen consumption rates (OCR) and extracellular acidification rates (ECAR) analysis, which showed also a reduction of oxidative phosphorylation and ATP production (Fig. 2c). To confirm the involvement of TRPM7 in cancer metabolism, qPCR results illustrated a down regulation of three important enzymes in KO cell line: hexokinase II (HK2), gamma enolase (ENO2) and pyruvate dehydrogenase kinase 1 (PDK1) (Fig. 2 d), which are involved in glycolysis pathway [83, 84]. The RNA-seq analysis highlighted the effector that regulates the TRPM7 glycolytic metabolism axis: SLC2A3, which encodes GLUT3. GLUT3 has an important role to deliver glucose to parenchymal brain cells [85] and it is hypoxia-responsive; for this reason, it is considered a hallmark for tumor vascularization and growth [86, 87]. The study conducted by Wu et al. demonstrated that in KO-TRPM7 cell lines, the transcript levels of SLC2A3 were very low in vivo(Fig. 2e-f) [6]. The data suggested that the SCL2A3-TRPM7 axis controls cellular glycolysis through Ca^2+^ signaling. Indeed, Ca^2+^ influx influenced SLC2A3 translocation through Ca^2+^-induced calcineurin activation. This axis was able to regulate the glucose pathway due to the ability of SCL2A3 to bind cAMP response element-binding protein (CREB), which is regulated by the CRTC2 cofactor. The status of CRTC2 as a major target of TRPM7-calcineurin was investigated, revealing that TRPM7 activation increased CREB phosphorylation and CRTC2 nuclear translocation, an effect that was blocked by the calcineurin inhibitor [11]. Overall, the results elucidated that targeting TRPM7 could modulate the glycolytic cancer pathway.

##### Ovarian cancer

Ovarian cancer is the deadliest gynaecological cancer. GLOBOCAN report revel that it affects approximately 2.5% of the female population. 207,000 women die each year, and it is estimated that the annual number of cases will increase by more than 40% by 2040. Survival rates depend on the stage at diagnosis. Early detection of the disease is challenging due to the lack specific symptoms. Often the diagnosis is made when the tumor is high grade, this is the reason why it is called “silent killer” [88]. Nowadays, the gold standard for ovarian cancer treatment consists of surgery followed by platinum-based chemotherapy. Due to the difficulty to prevent and to diagnose the tumor in the early stage, there is the necessity to explore alternative and more precise targets to offer hope to patients [89].

The important role of TRPM7 in metabolic reprogramming, especially in glycolytic pathway was also explored in the ovarian cancer [12]. In this recent study, Chen and colleagues evidenced how TRPM7 was overexpressed is ovarian cancer tissues, and it was positively correlated with glycolysis related genes, such as HK2, PDK1 but negatively with isocitrate dehydrogenase 3 (IDH3B) and ubiquinol-cytochrome c reductase core protein 1 (UQCRC1) (Fig. 2 g), two enzymes that are known to be involved in the OXPHOS pathway [68, 69, 90, 91]. To validate the primary role of TRPM7 in cancer metabolic reprogramming, the channel was silenced. Western blot analysis and real time PCR confirmed a decrease in glycolytic related genes and an increase of OXPHOS genes (Fig. 2 h-i). In addition, research has shown that TRPM7 regulates the hypoxia-inducible factor 1α (HIF-1α)-driven pathway by activating AMPK, which supports cancer progression and metastasis [65], by facilitating the formation of abnormal blood vessels that stimulate VEGF [66]. The study demonstrated that TRPM7 silencing promoted AMPK activation and decreased HIF-1α protein to shift preferred glycolysis to OXPHOS. Furthermore, Compound C (CC) AMPK inhibitor led to an increase in glycolysis markers like glucose uptake and lactate generation; however, these effects were mitigated by silencing HIF-1α (Fig. 2l, m). On the other hand, metformin-activated AMPK decreased glycolysis; this effect was countered by overexpressing HIF-1α. The Subsequently, we investigated how the silencing of TRPM7 affected AMPK activation, the expression of HIF-1α, OXPHOS, and glycolysis in ovarian cancer cells. Following the activation of AMPK through the administration of metformin, an increase in IDH3B and UQCRC1 was observed. However, this increase was subsequently reversed through treatment with CC, which was employed to inhibit AMPK. Furthermore, the silencing of HIF-1α led to a significant reduction in the levels of both IDH3B and UQRC1(Fig. 2n-o) [12].

In conclusion, these findings suggest a regulatory mechanism in which TRPM7 modulates cellular metabolic shifts in ovarian cancer by influencing the AMPK/HIF-1α signaling pathway, highlighting its role as a key mediator in metabolic reprogramming. Indeed, TRPM7 silencing enhanced AMPK activation, facilitating the ubiquitination and proteasomal degradation of HIF-1α, thereby attenuating HIF-1α-driven glycolytic flux and promoting a metabolic shift towards oxidative phosphorylation. The suppression of ovarian cancer cell proliferation and tumorigenicity is attributable to metabolic reprogramming, which elucidates the potential role of channel modulation in disrupting the metabolic plasticity that is essential for tumor growth.

##### Hepatocellular carcinoma

Hepatocellular carcinoma (HCC) is a major healthcare challenge in terms of incidence and cancer-related mortality. It is the sixth most diagnosed tumor and the third most common cause of cancer death. It is estimated that new cases will increase by more than 50% in 20 years [70, 92]. The early detection of HCC remains a significant clinical challenge, primarily due to the limited sensitivity of current surveillance tools such as liver ultrasound and serum α-fetoprotein [93]. Whilst there is encouraging evidence in support of the potential clinical utility of novel biomarkers and techniques, their full clinical potential is yet to be determined. Advancements in imaging technology have enhanced non-invasive diagnostic capabilities, thereby reducing the necessity for biopsy procedures. However, in certain instances, tissue confirmation remains essential for the diagnosis of atypical lesions [94]. As an alternative, liquid biopsies are emerging as a potential tool for safer, non-invasive tumor profiling [95]. Although chemotherapy plays an important role against this disease, its efficacy is limited due to drug resistance phenomena and insufficient selectivity of the drug against liver cells [96]. In the last decade, research has improved the therapeutic strategy to increase patient survival thanks to monoclonal antibodies and tyrosine kinase inhibitors [72, 97]. The significance of this tumor for society is such that there is a need to discover new biomarkers and therapeutic targets.

**Fig. 3:**
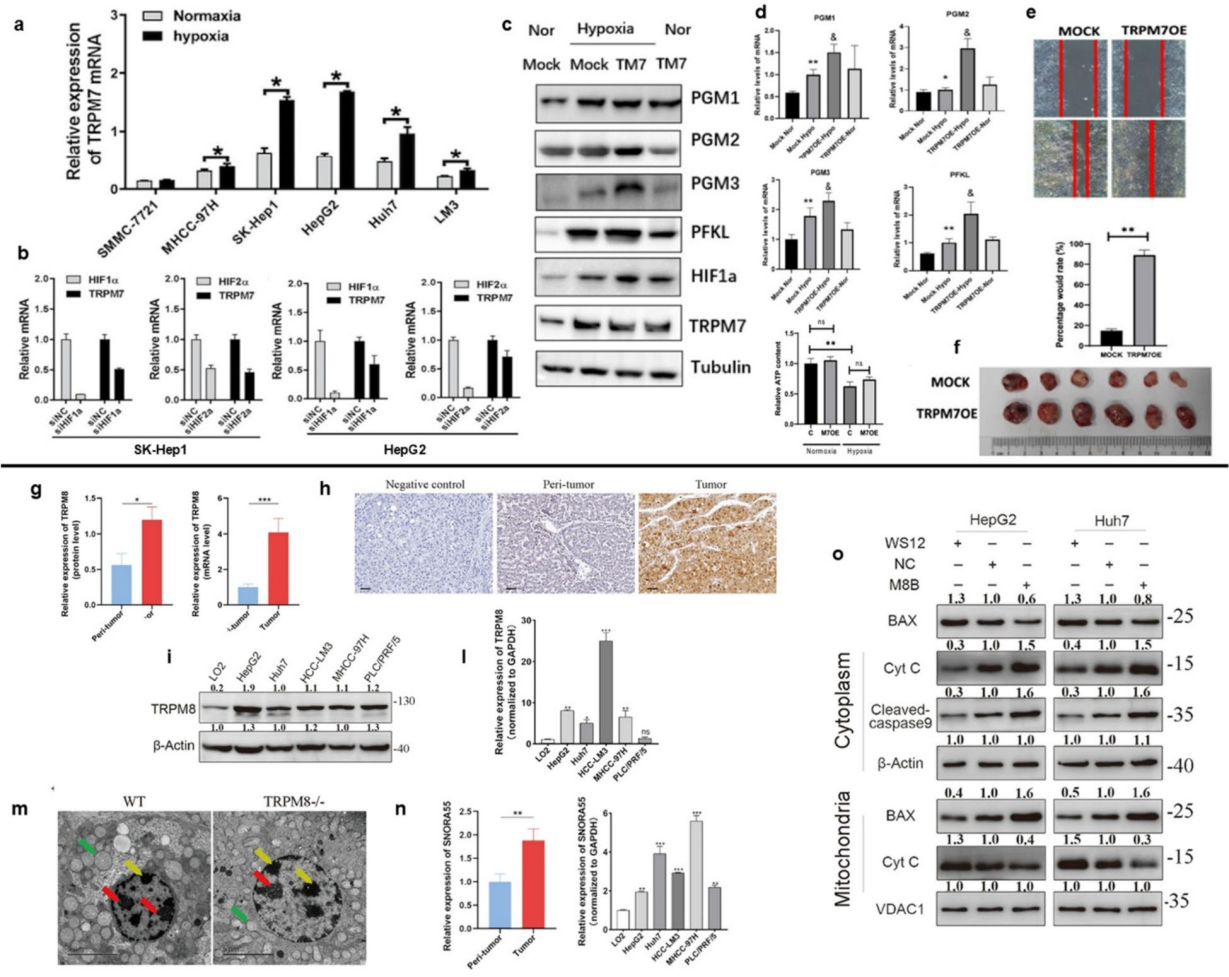
Role of *TRPM7* (**a**-**f**) and *TRPM8* (**g-o**) in metabolic reprogramming in Hepatocellular carcinoma. **a** The upregulation of TRPM7 expression in hypoxic HCC cell lines. In six HCC lines, 1% O_2_ treatment resulted in an increase in TRPM7 mRNA expression across a 24-h period. *, *p* < 0.05. **b** mRNA expression of HIF-1α and HIF-2α in SK-hep1 and HepG2 cells transfected with siRNA targeting HIF-1α or HIF-2α. **c-d** qPCR, Western blot and ATP detection of PGM1, PGM2, PGM3, and PFKL in Huh-7 cell line. *p < 0.05. **e–f** TRPM7 accelerated the growth of HCC. Overexpression of TRPM7 in Huh7 cells promotes the rate of wound healing (**e**) and tumor growth in nude mice (**f**) *, *p* < 0.05 [from a to f, *Copyright:xxx* [98]]. **g** TRPM8 was examined through qPCR in 20 samples of human HCC tissues. P tissue from tumors, T tissue from tumors. *p < 0.05, **p < 0.01, ***p < 0.001. **h** TRPM8 immunohistochemistry staining in 78 more human tissues. Bar scale: 50 µm. **i-l** TRPM8 was detected by Western blotting and qPCR in five HCC cell lines and the normal liver cell line L02. The data were expressed as the mean ± SEM. **m** TEM representative images. Red arrows indicate nucleoli, green arrows mitochondria, and yellow arrows chromatin. **n** qPCR was used to determine the expression level of SNORA55 in HCC and peritumor tissues. *p < 0.05, **p < 0.01, ***p < 0.001. **o** Western blotting was utilized to determine the presence of cleaved-caspase 9, Cyt C, and BAX after cytoplasmic and mitochondrial proteins were extracted. Internal controls included β-Actin in the cytoplasm and VDAC1 in the mitochondria [from g to o, *Copyright:xxx* [102]

This is the background to a very recent study published in 2024 by Zhao et al. which examined how TRPM7 can alters glucose metabolism in HCC [98]. The researchers firstly compared the expression of TRPM7 in cell lines cultured under normoxic and hypoxic conditions, revealing that the ion channel was highly expressed in hypoxic conditions across seven different HCC cell lines (Fig. 3a). The mRNA expression silencing HIF-1α and HIF-2α to identify which HIF subunit was directly controlled by TRPM7. The results clarified that HIF-1α was the direct target of TRPM7 (Fig. 3b), affecting the expression of 6-phosphofructokinase (PFK) and phosphoglucomutases (PMGs). It is well known that PFK and PMG are key enzymes involved in glycolysis [99–101].

The mRNA expression showed that PFK, PGM1, PGM2 and PGM3 were upregulated under hypoxic conditions (Fig. 3c-d) [98]. These data confirmed that metabolic remodeling is driven by TRPM7, which was also involved in migration and tumor proliferation. In fact, its knockdown resulted in reduced cell viability and migration capacity in vivo mice tumor (Fig. 3e-f). HIF-1α directly regulates the transcription of TRPM7, which results in the reprogramming of glycolytic metabolism and fosters the proliferation and metastasis of HCC both in vitro and in vivo*.*

#### TRPM8

##### Hepatocellular carcinoma

In the complexity of HCC, also TRPM8 could fulfil a special role. Within this framework, Fu et al. discovered that TRPM8 plays a strong oncogenic role in HCC by causing nuclear and mitochondrial dysfunction in a SNORA55-dependent manner [102]. TRPM8 overexpression correlated positively with tumor size, stage, and multiplicity in various patient tissues (Fig. 3g-h), and that the channel was overexpressed in various liver cancer cell lines compared to healthy cell lines (Fig. 3i-l). The carcinogenic role of TRPM8 has also been demonstrated in vivo. TRPM8-deficient mice exhibited livers with fewer nucleoli and smaller mitochondria compared to the wild type group, accompanied by chromatin morphology changes, as confirmed through immunohistochemical images by TEM and AgNOR analysis (Fig. 3m). To explore the TRPM8 involvement in HCC cancer progression channel knockout was fundamental. Indeed, KO-TRPM8 also determined a modification in the chromatin compartment and, subsequently, the gene expression. The pivotal role in this molecular mechanism was driven by small nucleolar RNA, which have been associated with the emergence of multiple cancer, including non-small lung cancer (SNORD33, SNORD66, and SNORD76), breast cancer (SNORA73A, SNORA73B, and SNORA74A), and prostate cancer (snoRA SNORD78) [103]. Fu et al., detected that the most important role is played by SNORA55, which is localized either in the nucleus and cytoplasm and positively correlated with TRPM8 [71]. The overexpression was observed in HCC tissues and cell lines (Fig. 3n). SNORA55 knockdown dramatically reduced colony formation, proliferation, and induced apoptosis, which is consistent with the involvement of TRPM8 in HCC. SNORA55 knockdown also reduced ATP content and OCR, it also facilitated the release of Cyt C from mitochondria into the cytoplasm, leading to cell death (Fig. 3o). This research elucidated that SNORA55 migration is involved in a mitochondrial molecular mechanism, interacting with ten proteins, particularly, ATP synthase components, such as ATP5A1 and ATP5B. Indeed, SNORA55 knockdown dramatically reduced the expression levels of ATP5A1 and ATP5B in TRPM8 overexpressed HCC cells. Taken together, the findings regarding TRPM8 and SNORA55 suggest that they could serve therapeutic targets for modulating hepatocarcinogenesis [102].

## TRP channels: sensors of tumor processes mediated by oxidative stress

Recent investigations have enlightened that TRP channels have been implicated in both promoting and mitigating oxidative stress damage, depending on the specific channel involved and its location within tissue [14]. Oxidative stress is a physiological condition characterized by an imbalance between the production of ROS and the body's ability to detoxify these reactive intermediates or repair the resulting damage [104]. Excessive production of ROS has been demonstrated to induce substantial damage to cellular components, encompassing lipids, proteins and DNA. This phenomenon has been associated with the aetiology of various diseases, with cancer being a prominent example [105].

The interplay between cancer and ROS is a complex phenomenon that has been extensively investigated. ROS have been shown to exhibit a dual role in cancer, functioning as both promoters of tumor progression and inducers of cancer cell death [106]. This ambivalence is mediated through key molecular pathways. Moderate ROS levels have been shown to activate signaling cascades such as PI3K/Akt, MAPK/ERK, and NF-κB, which enhance cell proliferation, survival, angiogenesis, and metabolic adaptation [107]. Conversely, elevated ROS levels have been observed to trigger stress response pathways, notably the p53 and JNK pathways, which can result in cell cycle arrest, apoptosis, or autophagy [108]. It is an established fact that cancer cells frequently upregulate antioxidant defences, such as the Nrf2/ARE pathway, in order to maintain redox homeostasis and evade ROS-induced toxicity [109]. From a therapeutic standpoint, the disruption of these adaptive mechanisms by either boosting ROS levels beyond the cellular threshold or inhibiting antioxidant responses presents a promising strategy [110].

Different studies have demonstrated that ROS influence diverse cancer signaling pathways, [106, 111, 112] including those responsible for cancer cell apoptosis, which is initiated through the activation of either the extrinsic or intrinsic pathway [113, 114]. Furthermore, ROS have been observed to regulate angiogenesis by modulating VEGF activity [115] and to affect drug response by altering drug efflux transporters [116].

In the complex and intricate interplay between oxidative stress and tumor progression, TRP channels exhibit a multifaceted role. It has long been known that oxidative stress may interfere with Ca^2+^ release from the endoplasmic reticulum (ER) by promoting or inhibiting Ca^2+^ sequestration by the Sarco-Endoplasmic Reticulum Ca^2+^-ATPase and Ca^2+^ release through inositol-1,4,5-trisphosphate receptors or ryanodine receptors [117–120]. More recent work found that ROS may exert a cytotoxic effect by directly or indirectly activating several TRP channels [117, 121, 122]. For example, TRPA1 channel promotes the upregulation of Ca^2+^-dependent, anti-apoptotic signaling pathways that enhance ROS tolerance [123]. TRPA1 expression is directly modulated by the oxidant-defense transcription factor nuclear factor erythroid 2-related factor 2 (NRF2) [14]. Similarly, TRPM2 also activated by oxidative stress, is crucial in mediating cellular responses to oxidative damage and promoting cell survival [124], but could also lead to ROS-dependent cell death [125, 126].

Recent studies suggest that the main channels involved in the oxidative stress-mediated cancer process are TRPML1 [15], TRPA1 [127–129], TRPM2 and TRPV1 [130].

TRPML1 is a non-selective cationic channel predominantly localized to the late endosomal and lysosomal compartments whose role in cancer progression has been recently acknowledged [19]. It can be activated by the lysosomal phosphoinositide, phosphatidylinositol 3,5-bisphosphate (PI(3,5)P(2)) [131], as well as by ROS [132] and mediates the release of multiple divalent cations, including Ca^2+^, Zn^2+^ and Fe^2+^, from the lysosome into the cytosol. As a result, TRPML1 is involved in the regulation of a wide range of cellular processes [133], such as lysosome fusion, reformation, exocytosis [134] and autophagy [135].

TRPA1 is the sole member of the mammalian TRPA1 subfamily and integrates chemical, physical, and thermal cues, being activated by a plethora of signals, including electrophilic and non-electrophilic agonists, cold and hot temperatures, and plasma membrane tension [123, 136]. Furthermore, TRPA1 is sensitive to ROS, ROS metabolites, as well as reactive nitrogen species (RNS), thereby emerging as a novel TRP sensor of redox signaling in mammalian cells [121, 123, 136, 137]. In addition, TRPA1 can be activated by many byproducts of lipid peroxidation, such as 4-oxo-nonenal, 4-hydroxyhexenal and 4-hydroxynonenal (4-HNE) [138, 139]. ROS may activate TRPA1 channels by oxidizing Cys621, Cys641, and Cys665 at the NH_2_ terminal of the channel protein [140]. TRPA1 is up-regulated in several malignancies, where it can either transduce ROS into pro-apoptotic Ca^2+^ signals or engage non-conventional anti-oxidant defense programs [123].

TRPM2 is a channel expressed in various tissues and organs, which plays an important role in Ca^2+^ homeostasis and oxidative stress. Excessive accumulation of ROS in the nucleus results in DNA damage, leading to the activation of poly-ADPR polymerase (PARP) and poly-ADPR glycohydrolase (PARG). Diffusion of ADPR to the cytoplasmic portion of TRPM2 at the plasma membrane triggers Ca^2+^ influx. The ability of TRPM2 to transduce ROS signals into Ca^2+^ signals positions it at the core of the cellular signaling network, where plays a critical role in buffering oxidative stress and maintaining Ca^2+^ homeostasis, which are essential for various cellular functions [141].

TRPV1 may engage a non-canonical antioxidant defense program through the Ca^2+^-dependent recruitment of the Ca^2+^/Calmodulin (CaM)-dependent Pyk2 [89, 123]. TRPV1, which is nonselective cation channels that prefer Ca^2+^ to Na^+^ ions [142]. It is responsive to a variety of chemical and physical stimuli, such as capsaicin and vanilloid analogues, low pH (pH < 6), endogenous agonists, such as fatty acids conjugated with amines, prostaglandins, and leukotriene B4, heat (temperature > 43 °C) and it is ubiquitously expressed [143, 144]. In addition, TRPV1 is also sensitive to cytosolic ROS [23, 121], which target Cys258 and Cys754 that are, respectively, located at the NH2- and COOH-tails of the human channel protein [145]. ROS-induced TRPV1-mediated Ca^2+^ has been shown to promote mitochondrial Ca^2+^ overload and caspase activation, thereby promoting apoptotic cell death in multiple cancer cell types [14, 146–148]. ROS-dependent TRPV1 activation in endothelial cells and circulating endothelial colony-forming cells may also promote tissue revascularization [144, 149].

### Significance of TRPML1, TRPA1, TRPM2 and TRPV1, in oxidative stress-mediated cancer processes

In the following subsections, we will examine the role of TRPML1, TRPA1, TRPM2 and TRPV1 in cancer processes mediated by oxidative stress in different tumor types (Table 2).

**Table 2:** Summary of recent findings on TRPML1, TRPA1, TRPM2 and TRPV1 channel and cancer oxidative stress

TRP Channel	Type of tumor	End-Point	Ref.
TRPML1	Metastatic melanoma	TRPML1 was upregulated in metastatic melanoma tissues and cell lines. This study correlated TRPML1 with lysosomal Zn^2+−^dependent necrotic cell death in cells treated with ML-SA, a channel inhibitor, while normal cells were spared. Cellular ATP depletion resulted from mitochondrial dysfunction induced by ML-SA treatment. While overexpression of TRPML1 in normal cells increased susceptibility to ML-SA, inhibition of TRPML1 prevented such cell death. ML-SA showed efficacy in further inhibiting tumor growth in in vivo models	[15]
TRPA1	Glioblastoma	The present study investigated the therapeutic role of TRPA1 in glioblastoma. The results demonstrate that channel activation increased the effect of TMZ, inducing the process of apoptosis in cells. The combined approach of TRPA1 activation and TMZ administration enhanced intracellular calcium influx, mitochondrial dysfunction and ROS production	[127]
TRPA1	Breast cancer	A non-canonical oxidative-stress defence through ion channel has been demonstrated. In breast cancer spheroids model evidenced a survival of inner cells experiencing ROS accumulation depended on ion channel, which enhanced Ca^2+^-dependent anti-apoptotic pathways. Furthermore, TRPA1 expression was regulated by NRF2, an oxidant-defence transcription factor, thereby offering an alternative protective mechanism against oxidative stress	[128]
TRPA1	Colorectal cancer	TRPA1 was upregulated in primary metastatic colorectal cancer cells. Furthermore, the study revealed that TRPA1 mediated H₂O₂-induced Ca^2+^ entry. The primary ROS activator of the channel was 4-HNE, which derived from lipid peroxidation. The combination of 4-HNE and H₂O₂ resulted in mitochondrial Ca^2+^ overload, leading to mitochondrial depolarisation and the activation of the apoptotic process via caspase-3/7	[129]
TRPA1, TRPM2, TRPV1	Colitis-associated cancer	TRPM2, TRPA1, TRPV1 were overexpressed in colon cancer cells. Treatment with SEB resulted in a reduction in the levels of caspases 3 and 9 in comparison with the control group. The administration of SEB led to a decline in mitochondrial membrane potential and ROS levels, thereby protecting colon tissue from oxidant damage	[130]

*Abbreviations*: *TRPML1* transient receptor potential mucolipin 1, *ML-SA* Mucolipin synthetic agonist, *ATP* Adenosine triphosphate, *TRPA1* transient receptor potential ankyrin 1 channel, *TMZ* Temozolomide chemotherapy drug, *ROS* oxygen-containing reactive molecules, *TRPM2* melastatin-related transient receptor potential cation channel 2, *TRPV1* vanilloid subtype 1 of transient receptor potential ion channels, *SEB Sambucus ebulus*, *NRF2* Nuclear factor erythroid 2-related factor 2, *4-HNE* 4-hydroxynonenal

#### TRPML1 in Metastatic melanoma

Melanoma is a cancer that affects melanocytes, cells that produce melanin in the basal layer of the epidermis. It is caused by different factors, and there has been a significant increase in new cases over the past 50 years. Skin melanoma accounts for 1.7% of global cancer diagnoses. [150] Thanks to new immunotherapies and a correct prevention path, we can identify and isolate the tumor in the early stages, reducing cancer-related deaths. The primary issue is that clinical diagnosis is predicated on the evaluation of morphological characteristics, rendering it inherently subjective and, at times, challenging for both dermatologists and general practitioners. This process generally entails visual inspection, frequently supplemented by dermoscopic evaluation of skin lesions [151]. In recent years, there has been a growing interest in the utilisation of artificial intelligence (AI) for the development of computer-assisted diagnostic tools with the objective of enhancing the accuracy of skin cancer detection [152].

The main challenge with this type of tumor is represented by metastasis, which is difficult to detect due to its poorly differentiated nature. To improve patient survival, it is so important to clarify cancer progression mechanisms and discover new potential targets [153, 154].

It has been demonstrated that TRP channels play a role in the oxidative stress observed in metastatic melanoma [155].

**Fig. 4:**
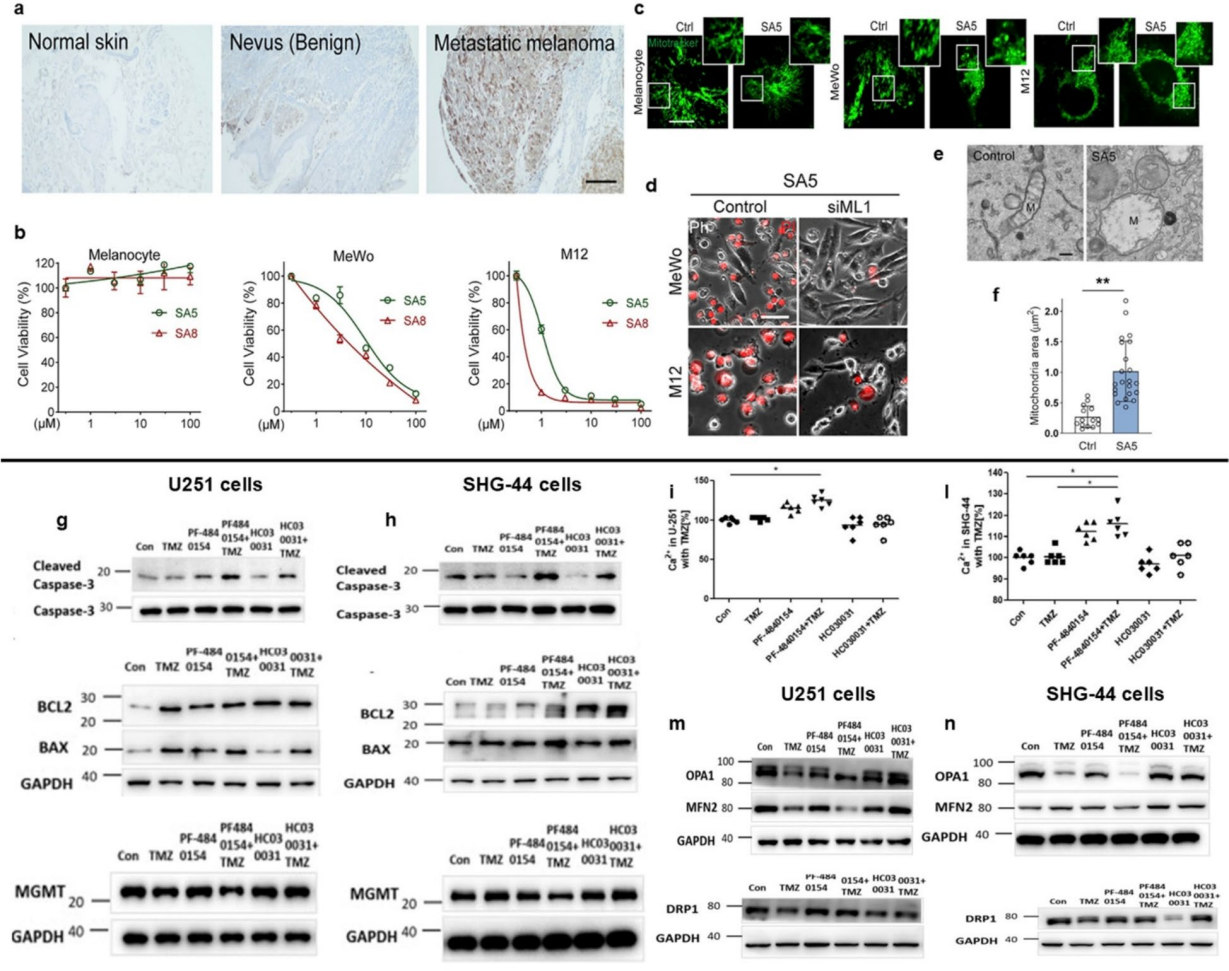
Involvement of *TRPML1* in metastatic melanoma (**a**-**f**) and *TRPA1* in glioblastoma cells (**g-n**) in cancer process mediated by oxidative stress. **a** An illustration of human tissue sections immunostained with an anti-ML1 antibody (brown DAB staining) from normal skin, nevus (benign tumor), and metastatic melanoma. 200 μm scale bar. **b** The effects of SA5 and SA8 on the dose-dependent viability of M12, MeWo, and normal melanocytes were assessed 24 h post-drug treatment using the CellTiter-Glo ATP assay. **c** Real-time imaging of MitoTracker-stained normal melanocytes (left panels, 60 min), MeWo (middle panels, 60 min), and M12 (right panels, 30 min) cells following treatment with DMSO (Ctrl) or SA5 (3 μM). Scale bar measures 10 μm. **d** PI-staining was performed on Ctrl or siML1-transfected MeWo and M12 cells with SA5 (3 μM) for a duration of 3 to 6 h. The composite images of phase contrast and PI are presented. The scale bar,25 μm. **e** Mitochondria in representative transmission electron microscopy (TEM) pictures of M12 cells treated for 30 min with either DMSO (Ctrl) or SA5 (3 μM). 200 nm is the scale bar. **f** A statistical study was conducted on the size of the mitochondria in control cells and SA5-treated cells **p < 0.01. [from a to f, *CC BY-NC-ND license* [15]]. **g-h** Apoptosis rate in response to TMZ and a TRPA1 agonist and inhibitor. In U251 and SHG44 cell lines, cotreatment with PF-4840154 and TMZ enhanced the activity of caspase-3 and caspase-9 as well as the protein expression of cleaved caspase-3. The impact of TMZ and a TRPA1 agonist inhibitor on intracellular Ca^2^⁺ concentrations in U251 cells was investigated using western blot analysis to measure the levels of Bcl-2 protein, BAX protein, and MGMT protein following TMZ or drug treatment in U251 cells. **i-l** In U251 cells and SHG-44 cells, cotreatment with TMZ and PF-4840154 raised the intracellular Ca^2+^ levels *p < 0.05. **m–n** Impact of TMZ and an agonist and inhibitor of TRPA1 on the expression of mitochondrial fission and fusion proteins. OPA1, MFN2, and DRP levels in SHG-44 and U251 cells following TMZ and a TRPA1 agonist and inhibitor therapy are analyzed through Western blot. GAPDH served as the internal reference [from g to n, *Creative Commons Attribution 4.0 International License* [127]

In this scenario, a recent work conducted by Du et al. improves the knowledge about the modulation of TRPML1 involved in metastatic melanoma using small molecules that were able to selectively induce metastatic melanoma cells in vitro and in vivo [15]. In their study, the authors first analyzed the upregulation of TRPML1 in metastatic melanoma cells compared to normal skin tissue (Fig. 4a). The role of TRPML1 was highlighted through its modulation, showing that two specific agonists decreased cell viability in skin cancer cell lines, while healthy melanocytes remained unaffected by the treatments (Fig. 4b). Using propidium iodide (PI) staining elucidated that the inhibitors ML-SA5 and ML-SA8 selectively induced necrosis-like cell death in melanoma cells (Fig. 4c). TRPML1 overexpression played a crucial role in ML-SA-induced cytotoxicity, as cell death was significantly reduced in ML1 knockdown cells. Additionally, the study proved evidence that this cell death was a Zn^2^⁺-dependent process, thanks to FluoZin-3-AM, a Zn^2^⁺-sensitive fluorescent dye. In MeWo and M12, two metastatic melanoma cell lines, vesicular compartments positive for LysoTracker exhibited the strongest FluoZin-3 signal, indicating that lysosomes are the main intracellular Zn^2^⁺ storage sites (Fig. 4d). These cells also showed a synergistic increase in ML-SA5-induced cytotoxicity, suggesting that Zn^2^⁺ release from lysosomes is the mechanism by which ML1 activation induces cell death in metastatic melanoma cells. Excess cytosolic Zn^2^⁺ is known to induce mitochondrial damage, leading to a significant reduction in cellular ATP levels [10]. MitoTracker labelling uncovered that mitochondria in MeWo and M12 cells grew and fragmented, a phenomenon not observed in normal melanocytes (Fig. 4e-f). Taken together, these findings suggest that ML-SA compounds activate ML1, trigger the release of lysosomal Zn^2^⁺, and ultimately cause mitochondrial damage and ATP depletion [15]. Based on these insights, the data demonstrate that TRPML1 is overexpressed in metastatic melanoma cells and has the potential to serve as a therapeutic target. Indeed, pharmacological activation of TRPML1 has been demonstrated to induce rapid necrotic cell death by triggering lysosomal Zn^2^⁺ release, leading to mitochondrial damage and ATP depletion. This effect is selective for metastatic cells, contrasting with the slower anti-proliferative response observed with TRPML1 inhibition. Consequently, TRPML1 has the potential to function as both a prognostic marker and a promising target for the selective treatment of metastatic melanoma.

#### TRPA1

##### Glioblastoma

Glioblastoma (GMB) is the most common and aggressive primary brain tumor. Patients have a poor prognosis, with a survival rate is 1.2 years after the first diagnosis [156] This is due to complex genetic changes and the ability of glioma stem cells to exhibit high potential self-replication, which contributes to progression, aggressiveness, and tumor drug resistance. It has an incidence of 3.21 per 100,000 population. The huge problem in this type of tumor is related to early detection. Differential diagnosis depends on symptom duration, sequence, and anatomical location. Given the vague and non-specific presentation of most brain tumors, clinicians must also consider a broad spectrum of neurological disorders as possible or coexisting diagnoses [157]. The Stupp protocol is the gold standard for therapy. It involves surgical resection, followed by radiation and chemotherapy with Temozolomide (TMZ) [158, 159]. Despite significant scientific advances, an effective treatment capable of eradicating the disease and avoiding resistance phenomena remains elusive [160]. The exploration of glioblastoma progression remains an ongoing challenge. Consequently, the identification of mechanisms underlying drug resistance phenomena is also of crucial importance. TRPA1 is up-regulated in many cancer cell types and is regarded as one of the primary cancer cell redox sensors, being sensitive to H_2_O_2_, 4-HNE, 4-oxo-nonenal, and 4-hydroxyhexena [14, 123]. A paper that analyzes this subject in interesting detail has been published by Chen et al. which presented how facilitating TRPA1 activation in glioblastoma cells could be instrumental to overcome TMZ resistance [127]. Specifically, two different glioma cell lines, U251 and SHG-44 were treated with TMZ, in combination with or without a TRPA1 agonist (PF-4840154) or inhibitor (HC030031). The data showed that pretreatment of glioma cells with TRPA1 agonist increased pro-apoptotic protein (caspase-3/caspase-9, BAX) and decreased the level of anti-apoptotic protein (Bcl-2) compared to pre-treatment with the TRPA1 inhibitor (Fig. 4g-h). The researchers hypothesized that the key role is played by oxidative stress, analysing markers such as manganese superoxide dismutase (MnSOD), NAD(P)H quinone dehydrogenase 1 (NQO1), Heme oxygenase-1 (HO-1). When U251 cells were exposed to TMZ for 24 h, the mRNA expression of MnSOD was considerably higher than at the basal level, while NQO1 and HO-1 also rose, albeit not significantly. In U251 and SHG-44 cells pre-incubated with PF-4840154, the expression of MnSOD, NQO1 and HO-1 mRNA and glutathione (GSH) levels were reduced compared to cells treated with TMZ alone. Intracellular oxidative stress was crucial to drug response: TMZ and PF-4840154 synergistically increased intracellular ROS levels, while HC030031 decreased intracellular ROS levels. The link between TRPA1 and TMZ treatment was also evidenced by Ca^2+^ influx analysis: exposure to TMZ combined with PF-4840154 resulted in elevated intracellular Ca^2^⁺ levels in U251 and SHG-44 cell lines (Fig. 4i-l). Furthermore, combined treatment with TRPA1 agonist and TMZ induced mitochondrial dysfunction, leading to mitochondrial membrane damaged. WB results elucidated those cells treated with TRPA1 agonists exhibited decreased expression of optic atrophy protein 1 (OPA1) and mitofusins (MFN2) and increased the expression of Dynamin-related protein (DRP1) compared to the control group [127] (Fig. 4m-n). As it known, OPA1, MFN2 are the master regulator of mitochondrial fusion [161]; while DRP1 is a key player in mitochondrial fission [162].

In conclusion, the findings suggest that GBM cells can develop resistance to TMZ treatment by generating ROS, thereby diminishing the drug's therapeutic efficacy. Activation of the TRPA1 signaling pathway induces Ca^2+^ influx and oxidative stress, which drives cells toward apoptosis. These studies could serve as a foundation for strategies aimed at reducing TMZ resistance and enhancing drug sensitivity and cell death rates by harnessing TRPA1 activation.

##### Breast cancer

Breast cancer is the most common cancer and the leading cause of death in women worldwide. In 2020, there were over 2.3 million new cases and 685,000 deaths. By 2040, new cases are expected to increase by 40% [163, 164]. Common therapy strategies include surgery, radiation, chemotherapy, hormonal therapy, and targeted therapy, but these treatments have significant shortcomings, including side effects, drug resistance, and variable efficacy. The enormous impact this type of tumor has on healthcare has spurred scientific research to make enormous strides in improving patient survival and prevention pathways. Early detection is the best ally in the fight against this disease [165]. For this reason, the integration of AI in imaging techniques, such as digital breast tomosynthesis, not only improves diagnostic accuracy but also has a meaningful impact on patient outcomes. By increasing sensitivity without raising false positives, AI helps detect tumors earlier and more reliably, reducing the risk of missed diagnoses. This leads to earlier interventions, fewer unnecessary biopsies, and ultimately less anxiety and better prognosis for patients [166].

It is important to improve the knowledge about this cancer and discover new players involved. The role of TRP channels in breast cancer progression and proliferation could provide a new strategy to improve our knowledge of this complex tumor.

**Fig. 5:**
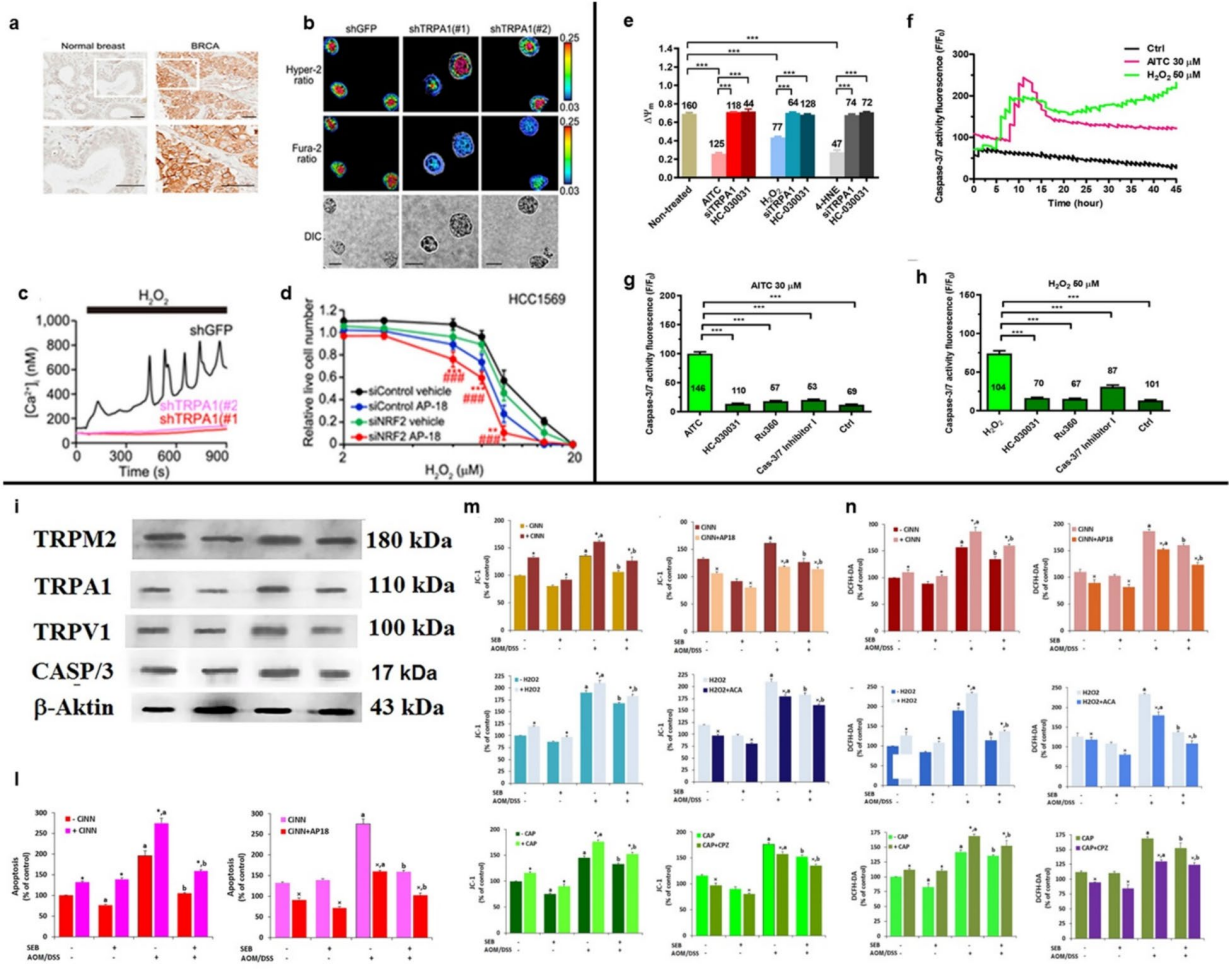
Involvement of TRPA1 in breast (panels a–d) and colorectal cancer (panels e–h), as well as the roles of TRPA1, TRPM2, and TRPV1 in colitis-associated cancer (panels i–n), during tumor progression driven by oxidative stress. **a** Immunocytochemical staining showing TRPA1 expression in normal versus breast tumor tissues. Scale bar = 50 μm. **b** Calcium influx response triggered by 10 μM H₂O₂ in HCC1569 cells. ***p < 0.001. **c** Representative images of Hyper-2 and fura-2 fluorescence ratio in day-5 HCC1569 spheroids transduced with either shTRPA1 or shGFP. Scale bar = 50 μm. **d** Relative live cell counts of HCC159 cells following a 72-h exposure to H₂O₂ with or without 10 μM AP-18. Data represent means ± SD from three independent experiments performed in duplicates. *p < 0.05, **p < 0.01, ***p < 0.001 compared to siControl cells treated with AP-18; ###*p *< 0.001 compared to siNRF2 cells with vehicle treatment [panels a–d, Copyright: xxx [128]]. **e** Mean ± SE measurements of mitochondrial membrane potential (∆Ψm) under control and various treatment conditions. ***p < 0.001. **f** Changes in CellEvent™ fluorescence indicating caspase-3/7 activity in control and after treatment with AITC (30 µM) or H₂O₂ (50 µM). **g** Quantification of CellEvent™ fluorescence intensity under the following conditions: Control; AITC (30 µM, 6 h); AITC + HC-030031 (30 µM, 30 min); AITC + Ru360 (5 µM, 30 min); AITC + Caspase-3/7 Inhibitor I (20 µM, 30 min). ***p < 0.001. **h** Quantitative analysis of CellEvent™ fluorescence intensity after treatment with: Control; H₂O₂ (50 µM, 6 h); H₂O₂ + HC-030031; H₂O₂ + Ru360; H₂O₂ + Caspase-3/7 Inhibitor I. ***p < 0.001 [panels e–h, Creative Commons Attribution 4.0 International License [129]]. **i** In a mouse model of AOM/DSS-induced tumors, administration of Sambucus ebulus L (SEB, 100 mg/kg/day) led to decreased expression of TRP channels and caspase-3 (CASP/3), with β-actin as loading control. **l** Colonic tissues from mice with colorectal cancer exhibited reduced apoptosis triggered by TRPA1 stimulation; treatment with the TRPA1 agonist (CiNN) enhanced apoptosis, whereas the antagonist reduced it. *p ≤ 0.05. **m** TRP agonist treatments elevated mitochondrial membrane potential (miPOT) in colon cells of AOM/DSS-induced colorectal cancer mice, with SEB modulating this effect (mean ± SD, *n* = 3). *p ≤ 0.05. **n** TRP agonists also increased cytosolic reactive oxygen species (cyROS) production in colon cells from the same model; SEB administration mitigated this increase (mean ± SD, *n* = 3). In the AOM/DSS group, cyROS levels were significantly elevated, while SEB treatment reduced these levels. *p ≤ 0.05 [panels i–n, Copyright: xxx [130]]

On this basis, the work carried out by Brugge and co-workers, showed that TRPA1 promoted upregulation of Ca^2+^-dependent anti apoptotic pathways to promote ROS resistance response [128]. Using The Cancer Genome Atlas Analysis (TCGA) and immunocytochemistry analysis evidenced that TRPA1 was overexpressed in breast cancer (Fig. 5a). Subsequent, the study investigated the effect of this channel in response to one of the most important ROS involved in cancer pathophysiology. Treatment of cells with H_2_O_2_ increased the intracellular Ca^2+^ concentration and the oscillatory Ca^2+^ response was suppressed by TRPA1 knockdown, demonstrating that this ion channel is the main player in the H_2_O_2_ response. The results were also confirmed in a more complex cell culture system, such as tumor spheroids, in which there was an accumulation of ROS, especially in the inner part, which determined an increase in intracellular Ca^2+^ concentration that was suppressed by TRPA1 inhibitor (AP-18) or by *N*-acetyl-l-cysteine (NAC) treatment (Fig. 5b-c). Because the cells in Matrigel were deprived of ECM signals, the authors suggested that this ion channel was involved in cell survival thanks to its ability to promote anchorage independent oxidative stress defense mechanism. Consistently, detachment, as well as H_2_O_2_ treatment, induced the expression of ROS-neutralizing genes, including NQO1 and glutamate cysteine ligase (GCLM), through NRF2 [128]. It is well documented that NRF2 upon activation, translocated to the nucleus and binds to the antioxidant response elements (ARE) of target gene promoter regions, such as NQO1 and GLCM [167]. The key role of NRF2 was underlined thanks to its knockdown. In HCC1569 cells, the inhibition of either TRPA1 or NRF2 led to suppressed cell survival (Fig. 5d). Additionally, the reduction in cell survival was greater when both NRF2-mediated programs were inhibited; suggesting that oxidative stress defense in this cell line is mediated separately by TRPA1-dependent and TRPA1-independent programs [128].

##### Colorectal cancer

Colorectal cancer (CRC) is the third most diagnosed tumor worldwide and the second most common cancer death cause. By 2040, it is projected that 3.2 million new cases will be diagnosed, and 1.6 million patients will have a fatal prognosis [168]. The risk factors vary, and the cancer is characterised by its heterogeneity in terms of symptoms, pathological and molecular features [169]. Early detection of CRC is vital but remains challenging. Screening methods are either noninvasive, like fecal tests, which are patient-friendly but less accurate, or invasive, like colonoscopies, which are highly reliable but less accessible. To address these challenges, ongoing research is exploring more advanced, accurate, and accessible screening technologies [170].The gold standard treatment include surgery, intervention, followed by radiotherapy and chemotherapy. However, drug resistance remains a significant challenge, mainly due to the limited understanding of CRC mechanisms. Therefore, researchers are discovering new targets to improve therapeutic strategies, with TRPA1 emerging as a promising ally thanks to its involvement in oxidative stress regulation.

In this scenario, a recent study by Faris et al. reported that TRPA1 protein is up regulated in primary cultures of metastatic CRC (mCRC) cells as compared to non-neoplastic cells [129]. Consistently, the selective electrophilic agonist, allyl isothiocyanate (AITC), evoked a robust intracellular Ca^2+^ overload [171], which may be regarded as a hallmark of pro-apoptotic Ca^2+^ elevations in mCRC cells, but not in their non-neoplastic counterparts [129]. AITC-evoked increased in [Ca^2+^]_i_ was suppressed by the removal of extracellular Ca^2+^ or when TRPA1 was inhibited either pharmacologically with HC-030031 or genetically via a selective small interfering RNA (siRNA) [129]. Furthermore, high concentrations of H_2_O_2_ (50 μM), which are in the same range that has been reported in the cancer microenvironment [172], also evoked a sustained increase in [Ca^2+^]_i_ in mCRC cells that was sensitive to the removal of extracellular Ca^2+^ and the abrogation of TRPA1 signaling. H_2_O_2_-dependent TRPA1 activation, in turn, led to mitochondrial Ca^2+^ overload, mitochondrial depolarization and caspase-3/7 activation (Fig. 5e-h), thereby causing mCRC cell death [129]. Intriguingly, H_2_O_2_-evoked robust Ca^2+^ signals were inhibited by deferoxamine, which interferes with H_2_O_2_ degradation into the hydroxyl radical (OH^•^) and lipid peroxidation [173]. In line with this, 4-HNE, which originates from the oxidation of omega-6 polyunsaturated fatty acids within the plasma membrane [128], evoked TRPA1-mediated prolonged Ca^2+^ signals, mitochondrial Ca^2+^ overload and mitochondrial depolarization in mCRC cells, thereby supporting the view that 4-HNE is the primary ROS responsible for TRPA1 activation [129]. In general, the results indicate that activating TRPA1 might be an encouraging treatment strategy to make mCRC cells more responsive to oxidative stress, potentially in conjunction with pro-oxidant treatments.

#### TRPA1, TRPM2 and TRPV1 in Colitis-associated cancer

Colitis-associated cancer (CAC) is the most important complication of a bowel inflammatory process. It is a subtype of colorectal cancer that occurs in both men and women equally [174] While there is still uncertainty around the exact incidence of this tumor, the risk factors are well established. These include age, inflammation, disease severity, and family history of colorectal cancer [175]. Although endoscopic tools and diagnostic methods have advanced significantly in recent years, early detection of the cancer remains difficult due to the presence of inflamed mucosa in ulcerative colitis and the varied appearance of lesions [176].The conventional therapies include drugs that modulate the activity of inflammatory cells. 5-Aminosalicylic acid (5-ASA, mesalazine) is an effective medication that reduces intestinal inflammation and is used for chemoprevention. Mesalazine and endoscopic surveillance are the only effective strategies for treating this tumor, which has a poor prognosis for patients [177]. Understanding the role of different players in inflammatory processes is crucial if the goal is to develop a specific strategy to keep this tumor under control.

One of the most recent studies conducted by Kaya et al. unveiled how overactivation of different TRP channels, such as TRPA1, TRPM2 and TRPV1, caused oxidative stress, apoptosis and, consequently, cell death in mice colorectal cancer [130]. The study specifically investigated the preventive properties of a plant extract on apoptosis and ROS generation. The researchers induced colitis-associated colon cancer in mice and showed that the expression levels of the three TRP channels (TRPA1, TRPM2, TRPV1) were overexpressed (Fig. 5i). To examine the apoptosis process, the colorectal cancer cells from mice were analyzed (Fig. 5l). The results indicated increased apoptosis in the control group, while cells treated with *Sambucus Ebulus* L. (SEB), which is an antioxidant plant extract able to induce anticancer immunotoxins, exhibited reduced apoptosis [178].

There was also an increase of apoptosis after administration of ion channels agonists: cinnamaldehyde (TRPA1), H_2_O_2_ (TRPM2) and capsaicin (TRPV1). A reduction of apoptosis was observed after antagonists’ administration: AP18 (TRPA1), ACA (TRPM2), and capsazepine (TRPV1). Notably, pretreatment of mice with SEB decreased the level of caspase 3 and 9 compared to the control group. The protective role of SEB was also illustrated in oxidative stress modulation: the pretreatments diminished both the levels of mitochondrial membrane potential (myPOT) through JC-1, and mitochondrial ROS (cyROS) by DCFH-DA (Fig. 5m-n). The results confirmed what was seen with the apoptosis analysis: SEB treatment had a negative effect on colon cancer but shielded the healthy colon from oxidative stress and apoptosis, protecting it by preventing oxidant action [130]. Therefore, it may be useful to continue exploring the mechanisms underlying SEB action in order to translate these effects into the clinic and improve patient survival.

## Molecular Crosstalk Between TRP Channels, Metabolism, and Oxidative Stress in Tumor Progression

The involvement of TRP channels in cancer highlights their crucial role in orchestrating metabolic reprogramming and oxidative stress responses through diverse molecular pathways. In this section, we will specifically focus on molecular pathways affected by TRPs channels in tumor modulation (Table 3).

**Table 3:** Summary of molecular pathways affetcted by TRP channels in tumor modulation

TRP Channel	Molecular Pathways	Ref.
TRPM7	↑ Ca^2^⁺ via TRPM7 activates calcineurin, which dephosphorylates CRTC2, enabling its nuclear translocation. In the nucleus, CRTC2 binds phosphorylated CREB (p-CREB), activating transcription of SLC2A3 (GLUT3). This increases glucose uptake, aerobic glycolysis (↑ ECAR, lactate), and cellular ATP. Pharmacological activators/inhibitors validate the pathway: TRPM7 → Ca^2^⁺ → calcineurin → CRTC2/CREB → SLC2A3	[11]
TRPM7	TRPM7 knockdown leads to increased phosphorylated AMPK (p-AMPK). Active AMPK triggers ubiquitination and proteasomal degradation of HIF-1α, reducing its level. Consequently, glycolytic enzymes (HK2, PDK1, PKM2) decrease, while oxidative phosphorylation enzymes (IDH3B, UQCRC1) increase. Metabolically, cells show ↓ ECAR, ↑ OCR, higher ATP and ROS — collectively suppressing ovarian cancer cell proliferation	[12]
TRPM7	Under hypoxic conditions, TRPM7 expression is upregulated HCC. Though the downstream signaling isn't fully mapped, TRPM7 promotes glycolytic reprogramming and HCC progression via mechanisms likely analogous to the Ca^2^⁺/CRTC2/CREB and HIF-1α/AMPK axes	[98]
TRPM8	TRPM8 overexpression triggers epigenetic changes upregulating SNORA55, an H/ACA box snoRNA. SNORA55 translocate to the mitochondria, where it interacts with ATP5A1 and ATP5B (components of ATP synthase), disrupting mitochondrial function (e.g., respiratory dynamics and ATP production). This mitochondrial dysregulation contributes to enhanced proliferation, migration, and apoptosis resistance in HCC cells	[102]
TRPML1	Lysosomal Zn^2^⁺ release via TRPML1 triggers mitochondrial Zn^2^⁺ accumulation, leading to mitochondrial dysfunction (↓ membrane potential, ↑ ROS) and non-apoptotic cell death. This process bypasses caspase activation. Mitochondria-lysosome crosstalk and secondary Ca^2^⁺ signaling amplify stress responses. Pharmacological modulation (ML-SA1, bafilomycin A1, Ru360) confirms the pathway: TRPML1 → Zn^2^⁺ → mitochondria → ROS/Δψm loss → non-apoptotic death	[15]
TRPA1	Activated TRPA1 promotes mitochondrial fission via Drp1 recruitment, overcoming TMZ resistance in glioblastoma. TRPA1 activation leads to Ca^2^⁺ influx, enhancing mitochondrial fragmentation and increasing ROS. This sensitizes cells to chemotherapy. Pharmacological agents validate the pathway: TRPA1 → Ca^2^⁺ → Drp1 → mitochondrial fission → ↑ ROS → sensitization to TMZ	[127]
TRPA1	TRPA1, hijacked by cancer cells, enhances oxidative stress tolerance. Upon activation by ROS (e.g., H₂O₂), TRPA1 increases Ca^2^⁺ influx, which upregulates NRF2-dependent antioxidant transcription. This maintains redox balance and supports survival under stress. Genetic and pharmacological tools (shTRPA1, AITC, HC-030031) confirm the axis: TRPA1 → Ca^2^⁺ → NRF2 → antioxidant gene expression → oxidative stress resistance	[128]
TRPA1	TRPA1 activation by ROS in metastatic colorectal cancer cells triggers Ca^2^⁺ entry, mitochondrial dysfunction (↓ Δψm), and apoptosis via caspase-3/7. Sustained Ca^2^⁺ influx leads to elevated ROS and mitochondrial collapse. Antagonists (HC-030031) inhibit the pathway: TRPA1 → ROS → Ca^2^⁺ → mitochondrial dysfunction → caspase-3/7 activation → apoptosis	[129]
TRPA1, TRPM2, TRPV1	TRP channel stimulation in colitis-associated colon cancer induces oxidative stress and apoptosis. TRPA1 activation increases Ca^2^⁺ influx and ROS, leading to mitochondrial damage and activation of apoptotic pathways. Natural antioxidant *Sambucus ebulus L*. reverses these effects. Pathway: TRPA1/TRPV1 → Ca^2^⁺ → ROS → mitochondrial stress → apoptosis (↓ via S. ebulus)	[130]

*Abbreviations*: *TRPM7* transient receptor potential cation channel subfamily M member 7, *CRTC2* CREB Regulated Transcription Coactivator 2, *SLC2A3* Solute Carrier Family 2 Member 3, *GLUT3* Glucose transporter- 3, *KO* Knock-out, *AMPK* AMP-activated protein kinase, *HIF-1α* Hypoxia-Inducible Factor 1 alpha, *HK2* Hexokinase2, *ENO2* Enolase 2, *PDK1* Phosphoinositide-dependent kinase-1, *IDH3B* isocitrate dehydrogenase (NAD(+)) 3 non-catalytic subunit beta, *UQCRC1* ubiquinol-cytochrome c reductase core protein 1, *ROS* oxygen-containing reactive molecules, *ATP* Adenosine triphosphate, *HCC* Hepatocellular carcinoma, *TRPM8* transient receptor potential cation channel subfamily M member 8, *SNORA55* Small Nucleolar RNA, H/ACA Box 55, *TRPML1* transient receptor potential mucolipin 1, *ML-SA* Mucolipin synthetic agonist, *Δψm* mitochondrial membrane potential, *TRPA1* transient receptor potential ankyrin 1 channel, *Drp1* Dynamin-related protein, *TMZ* Temozolomide chemotherapy drug, *NRF2* Nuclear factor erythroid 2-related factor 2, *TRPM2* transient receptor potential cation channel subfamily M member 2, *TRPV1* vanilloid subtype 1 of transient receptor potential ion channels

Notably, TRPM7 has been implicated in promoting glycolysis and angiogenesis via upregulation of c-Myc and HIF-1α, key transcription factors that facilitate metabolic adaptation and vascular remodeling. In hepatocellular carcinoma, hypoxia-induced TRPM7 expression stabilizes HIF-1α and activates the PI3K/AKT pathway, enhancing glycolytic flux and tumor progression, while its silencing in ovarian cancer leads to AMPK activation and subsequent HIF-1α degradation through the PHD-VHL axis, thereby disrupting hypoxia-driven metabolic plasticity [11, 12, 95]. TRPM8 similarly supports tumor growth by modulating nuclear-mitochondrial communication; it induces SNORA55 expression, promoting mitochondrial biogenesis and hepatocellular carcinoma proliferation [99]. TRPML1, through lysosomal Zn^2^⁺ release, links ion-mediated stress to non-apoptotic cell death via mitochondrial depolarization in metastatic melanoma [15]. TRPM2, a redox-sensitive TRP channel, modulates cell survival and mitochondrial function in a ROS-dependent manner [124]. In breast cancer and colorectal cancer, TRPM2 activates NRF2 signaling to enhance resistance to oxidative stress [124, 125], while TRPA1 in glioblastoma alters mitochondrial dynamics and triggers apoptosis via increased fission, Ca^2^⁺ influx, and ROS production [126]. Additionally, in colitis-associated colon cancer, stimulation of TRPA1, TRPM2, and TRPV1 increases mitochondrial ROS and Zn^2^⁺ dysregulation, promoting apoptotic cell death, an effect mitigated by antioxidant treatment with *Sambucus ebulus L* [127]*.* Collectively, these findings reveal that TRP channels intersect with central pathways such as HIF-1α, AMPK, PI3K/AKT, NRF2, and mitochondrial signaling, underscoring their pivotal role in supporting tumor metabolism, redox balance, and therapeutic responsiveness.

## Harnessing TRP channels and nanotechnology for targeted cancer therapy

**Fig. 6:**
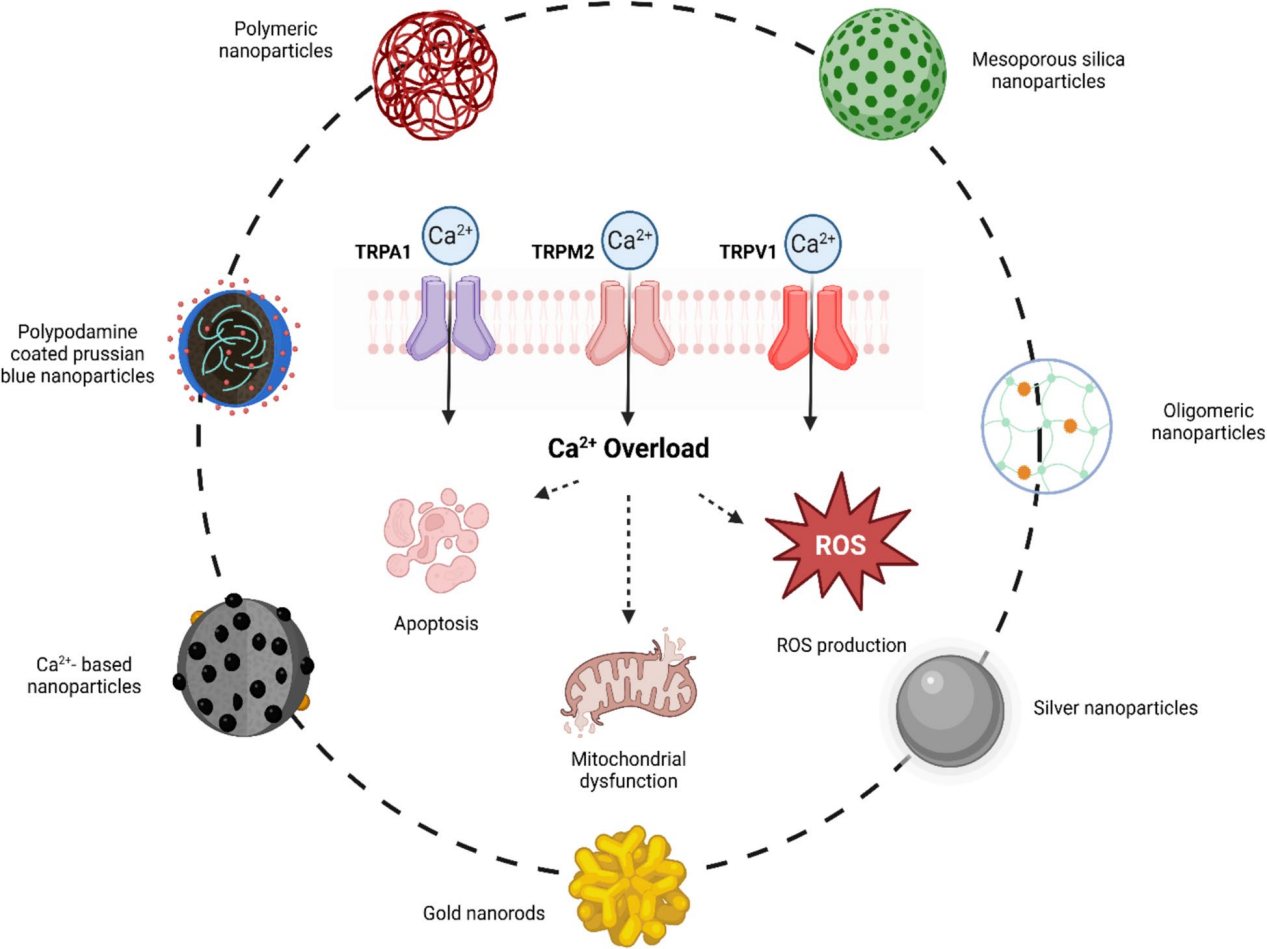
Biomedical Applications of Nanomodulators for [Ca^2+^]i Regulation. Several nanomaterials have been applied to modulate intracellular calcium levels by activating reactive oxygen species (ROS)-sensitive TRP channels, including TRPA1, TRPM2, and TRPV1. Upon stimulation, [Ca^2+^]i levels rise, leading to apoptosis

Recent advancements in nanotechnology and bioelectricity have considerably augmented the potential for innovative cancer therapies, offering novel approaches for both diagnostics and treatment [179]. The multifaceted function of TRP channels is emphasised by the pharmacological manipulation of ROS-sensitive TRP channels, which could provide an alternative targeted therapeutic strategy [180, 181]. Among the TRP channels, certain isoforms, such as TRPA1, TRPM2, and TRPV1, are particularly sensitive to ROS and contribute to disease progression by altering cellular signaling and promoting inflammation [5] (Table 4). The employment of nanomaterials to modulate the activity of ROS-sensitive TRP channels has emerged as a promising approach, however developing nanoparticle-based strategies for targeting TRP channels remains a challenging process (Fig. 6) [16]. To ensure both efficacy and safety, it is essential that several critical factors are given due consideration: firstly, the development of nanomaterials with high membrane affinity and spatial specificity, enabling their accumulation near TRP channels, either via direct molecular recognition (e.g. ligand-receptor interactions) or through engineered surface functionalities [165]. Also, the physicochemical characteristics of the nanoparticles, including surface charge and functional group density, are calibrated to ensure optimal equilibrium between cellular uptake efficiency, retention time at the membrane interface, and the reduction of off-target interactions [165]. Finally, the selection of an external stimulus (e.g., photothermal, magnetothermal, acoustic) must be appropriate with consideration for tissue penetration, spatiotemporal precision, and the potential for collateral damage resulting from local heating or reactive species generation [182].

**Table 4:** Summary of recent findings on TRPA1, TRPM2 and TRPV1 biomedical applications of Ca^2+^ signaling nanomodulators in oncology treatment field

TRP Channel	Type of nanosystem	End-Point	Ref
TRPA1	Polymeric nanoparticles	Conjugated polymeric nanoparticles were designed to specifically target TRPA1, which is overexpressed in breast cancer. After NIR light activation, nanoparticles were able to interfere with the defense mechanisms against oxidative stress and reduced the antiapoptotic responses in breast cancer cells	[183]
TRPA1	Mesoporous silica nanoparticles	Mesoporous silica nanoparticles were engineered to encapsulate a photothermal agent along with a thermal-sensitive nitric oxide donor. They were coated with hyaluronic acid, which acted as a targeting agent for tumor cancer cells. Under NIR exposure, the Ca^2+^ nanomodulators produced ROS that trigger TRPA1 activation. The photo-controlled release of Ca^2+^ influx facilitated an overload of Ca^2+^ in the cytoplasm inhibiting tumor progression	[184]
TRPM2	Oligomeric nanoparticles	Oligomer nanoparticles were developed to enhance TRPM2 activation, promoting calcium cascade signaling. Following NIR activation, there was a regulated production of ROS that suppressed early autophagy and further stimulated intracellular ROS production, ultimately resulting in mitochondrial dysfunction and the death of prostate cancer cells	[185]
TRPV1	Silver nanoparticles	Silver nanoparticles infused with 5-fluorouracil activated TRPV1 in colorectal cancer cells. The combined effect of the nanocarrier and 5-FU enhanced calcium signaling, leading to increased activation of caspase-3, −8, and −9, mitochondrial depolarization, lipid peroxidation, and ROS production, all of which contributed to cancer cell death	[186]
TRPV1	Gold nanorods	Gold nanorods have been shown to specifically bond to TRPV1 on ovarian and breast cancer cells. Thanks to their peculiar photothermal properties, gold nanorods have been demonstrated to evoke calcium influx, thereby determining increased apoptotic cell death and mitochondrial dysfunction	[187]
TRPV1	Calcium based nanoparticles	Fe-PDA@CaCO_3_ nanoparticles were synthesized to activated TRPV1 in hepatocellular carcinoma cell. When subjected NIR light, the nanoparticles caused a significant increasing in temperature, which activated TRPV1 channel and facilitated a substantial Ca^2+^ influx into the cell's cytoplasm. NPs interacted with H_2_O_2_, overexpressed in tumors, to generate ˙OH, thereby raising intracellular levels of ROS disturbing mitochondrial homeostasis and triggered the apoptosis pathway	[188]
TRPV1	Polydopamine coated hollow Prussian blue nanocages	PDA-hPBNCs linked to TRPV1 antibodies successfully attached to TRPV1-positive glioblastoma cancer cells and subsequently stimulated them through localized heating using NIR, resulting in an increased influx of Ca^2+^. The exposed hPBNCs in the acidic environment of tumor cells converted endogenous H_2_O_2_ into ˙OH via the Fenton reaction, thereby achieving CDT and enhancing cancer cell apoptosis	[148]

*Abbreviations*: *TRPA1* transient receptor potential ankyrin 1 cation channel, *NIR* near-infrared light spectrum, *ROS* oxygen-derived reactive species, *TRPM2* member 2 of the transient receptor potential melastatin subfamily, *TRPV1* member 1 of the transient receptor potential vanilloid subfamily, *5-FU* 5-fluorouracil, *Fe-PDA@CaCO3* alcium carbonate nanoshell mineralized ferric polydopamine nanoparticles, *NPs* nanoparticles, *H*_*2*_*O*_*2*_ hydroxide peroxide, *OH* hydroxyl radicals, *PDA-hPBNCs* Polydopamine coated hollow prussian blue nanocages, *CDT* chemodynamic therapy

In this context, a novel approach to tumor therapy has been suggested, namely the utilisation of near-infrared (NIR) light-responsive polymer nanoparticles (CPNs) conjugated with TRPA1 inhibitors [142]. This strategy has been identified as a promising avenue for breast cancer therapy, with the CPNs designed to absorb NIR light, thereby inducing localised hyperthermia within the tumor microenvironment. The generation of heat as a consequence of this process has been demonstrated to directly disrupt tumor cells and to trigger the activation of TRPA1. This, in turn, has been shown to promote apoptosis and inhibit cell proliferation, thus enhancing the therapeutic efficacy of the treatment. The combination of photothermal-induced tumor destruction and the modulation of TRPA1 channel activity resulted in a further potentiation of the anticancer effects, leading to a reduction in off-target effects and minimised damage to healthy tissues [183]. It has been demonstrated that TRPA1 could be modulated through nanostrategies in mammary, melanoma, ovarian and cervical cancer [143]. A photo-controlled Ca^2+^ nanomodulator was developed to leverage endogenous Ca^2+^ from two distinct sources for enhanced tumor therapy. The nanomodulator, which is loaded with indocyanine green (ICG), activates upon NIR light exposure, generating ROS that stimulated the TRPA1 ion channels. This activation facilitated the influx of Ca^2+^ ions from the extracellular environment into cancer cells, concurrently with the decomposition of BNN-6, a thermal-sensitive compound, releasing nitric oxide (NO). Consequently, a decreased in mitochondrial dysfunction and ATP was observed [184].

In addition, oligomer nanoparticles (CONPs) have been shown to manipulate ROS-sensitive TRPM2 channels within prostate cancer cells [144]. The nanoparticles have been designed to absorb NIR light and convert it into thermal energy, with a controlled release of ROS within the tumor microenvironment. The accumulation of ROS activated Ca^2+^ channels, which in turn induced mitochondrial dysfunction, leading to the demise of cancer cells [185].

Another strategy that has been explored was the synergistic effect of silver nanoparticles (AgNPs) in enhancing the anticancer properties of 5-fluorouracil (5-FU), a chemotherapy drug commonly used in the treatment of colorectal cancer, which is characterised by TRPV1 expression [145]. The impact of AgNPs on 5-FU's effects is twofold: they potentiated the effects of 5-FU and caused an upregulation of TRPV1 expression. This results in an increased influx of Ca^2+^ ions into cancer cells, leading to an accumulation of mitochondrial and intracellular ROS, the activation of caspases, and mitochondrial dysfunction. The AgNPs modulated TRPV1 activity, contributing to a more robust therapeutic response in comparison to 5-FU alone. This interplay between nanomaterials and Ca^2+^ signaling could be harnessed for more effective cancer therapies [186]. In addition, gold nanorods (GNRs) can be utilised to modulate TRPV1 activity. This innovative approach involved the combination of GNRs with NIR light to regulate membrane ion channels and activated apoptotic pathways in various cancer cells, including CHO and MCF-7 [146]. The study emphasised the distinctive photothermal properties of GNRs, which efficiently absorb NIR light and convert it into heat. The researchers have demonstrated that NIR-mediated heating of GNRs can trigger the activation of TRPV1, inducing an increase in caspase activation, mitochondrial dysfunction, and DNA fragmentation, all of which culminate in the targeted destruction of cancer cells [187].

Furthermore, evidence has emerged that the TRPV1 channel can be utilised to modulate hepatocellular carcinoma [147]. Calcium carbonate (CaCO_3_) nanoshells, mineralised with ferric polydopamine (Fe-PDA@CaCO3 NPs), have been engineered to facilitate tumor therapy through Ca^2+^-overload mechanisms. Upon reaching the tumor site, the CaCO_3_ shell, which is sensitive to pH changes, underwent rapid breakdown in the slightly acidic environment, resulting in the release of a substantial amount of free Ca^2+^. The Fe-PDA core, renowned for its remarkable capacity to convert light into heat, absorbs NIR light, inducing a substantial temperature rise. This elevated temperature activated the TRPV1 channel, leading to a substantial influx of Ca^2+^ into the tumor cells. In addition, the Fe-PDA core was able to interact with the H_2_O_2_ overexpressed in tumor cells, generating hydroxyl radicals (•OH). This reaction elevated intracellular ROS, which in turn inhibited the function of the Ca^2+^ efflux pump, resulting in a further increase in intracellular Ca^2+^ levels. The combined rise in Ca^2+^ and ROS has been shown to disrupt mitochondrial function, triggering the activation of the apoptosis pathway and effectively promoting tumor cell death [148].

The multifunctional nanoscale platform, based on polydopamine (PDA)-coated hollow Prussian blue nanocages (hPBNCs), was designed for targeted glioblastoma cancer therapy, integrating chemotherapy, chemodynamic therapy (CDT), and photothermal activation of TRPV1 channels [148]. The Prussian blue nanocages offered high drug-loading capacity and were responsive to NIR light. The nanoplatform utilised the Fenton reaction to generate ˙OH, inducing CDT, while the PDA coating functioned as a pH- and NIR-sensitive gatekeeper, preventing premature drug release while allowing specific targeting of TRPV1 channels on cancer cells. Upon NIR irradiation, TRPV1 activation triggered a calcium influx that led cancer cells to apoptosis [188].

These findings emphasise the necessity for further research to establish a connection between fundamental biological principles and clinical applications, thereby facilitating translational advancements in cancer care.

## Conclusions

There is growing evidence that TRP channels are involved in cancer progression and metastasis. This review underscores the expanding knowledge of TRP channels and their critical involvement in metabolic rewiring and oxidative stress in the promotion of cancer progression. From the recent literature, it was found that certain types of TRP channel are engaged in different types of cancers. Specifically, TRPM7 contributes to metabolic reprogramming in bladder, ovarian cancer and hepatocellular carcinoma and TRPM8 in hepatocellular carcinoma. Both TRPM7 and TRPM8 affect the glycolytic pathway, and while TRPM7 is also hypoxia responsive, the research highlighted that TRPM8 is further involved in chromatin organisation and mitochondrial functions.

In the second part of this review, we focused on recent studies that have highlighted the involvement of TRP channels in oxidative stress-mediated tumor progression. The most recent studies associate TRPML1 with Zn^2+^ lysosomal necrotic cell death in metastatic melanoma, TRPA1 with ROS accumulation and consequent mitochondrial dysfunction in glioblastoma, breast and colorectal cancer, and finally modulation of TRPM2 and TRPV1 activity in colitis-associated cancer with protection of colon tissue from oxidative damage.

In the last section, we highlighted the critical role of targeting nanosystems pathways via sensitive ROS channels, including TRPA1, TRPM2, and TRPV1, in the development of innovative therapeutic strategies. By harnessing the interplay between biological pathways, oxidative stress, and photodynamic therapy, this approach offers promising strategies for improving current treatment options.

TRP channels have been identified as novel therapeutic targets in oncology, with the potential to address significant challenges such as drug resistance and tumor heterogeneity. However, their clinical translation remains constrained by significant impediments, including their widespread expression in both healthy and malignant tissues, which gives rise to concerns regarding specificity and off-target effects.

It is recommended that future research must be concentrate on elucidating their complex roles within the tumor microenvironment, preferably through the 3D models that more accurately replicate in vivo conditions as demonstrated by recent studies. A TRPM8 channel inhibitor-encapsulated hydrogel has shown promise as a tuneable surface for bone tissue engineering by promoting osteogenic differentiation [189]. Similarly, hydrogels enabling controlled release of TRPV1 modulators have been employed to fine-tune osteoclastogenesis, highlighting the capacity to balance bone remodeling processes [190]. Imine-based nanofibrillar hydrogels with adaptable relaxation properties have been shown to activate TRPV4, enhancing cell mechanotransduction and promoting tissue regeneration [191]. Beyond skeletal applications, MMP-9-responsive hydrogels have been developed to aid diabetic wound healing by suppressing ferroptosis in endothelial cells, illustrating the versatility of hydrogel systems in targeting both structural and vascular components of tissue repair. Collectively, these studies underscore the potential of TRP-modulating hydrogels as dynamic platforms to study physiopathological conditions and these results demonstrated they could become good structure to better understand the role of these channels in 3D tumor microenvironment.

This review also underscores how targeting ROS and cancer metabolism could represent a promising and increasingly interconnected strategy in cancer therapy development. Despite this progress, the molecular mechanisms underlying ROS regulation and metabolic heterogeneity in cancer remain incompletely understood. The success of ROS and metabolism-targeted therapies will rely on patient stratification, improved and suitable experimental models, standardized methods for detecting specific ROS species. Personalized treatment approaches will be essential. From a clinical perspective, only a small number of trials are currently investigating TRP channels. TRPA1 is the focus of research in the context of oral cancer (NCT05024383). TRPV1 is the subject of investigation in relation to two specific health conditions: cancer pain (NCT04572776) and gastrointestinal tumor inflammation (NCT02666976). TRPC6 is being explored as a biomarker for cardiotoxicity in breast cancer (NCT05507879), while TRPV6 is currently in phase I trial for advanced ovarian cancers (NCT01578564) [5]. These preliminary studies, while limited in scope, underscore the necessity for rigorous preclinical validation and well-designed clinical trials.

Addressing these challenges will require an interdisciplinary effort that integrates molecular biology, pharmacology, bioengineering, clinical oncology and artificial intelligence. This approach will be pivotal in fully leveraging the potential of TRP channels as the next generation of targets in cancer therapy.

## Data Availability

No datasets were generated or analysed during the current study.
